# IL-10 attenuates OxPCs-mediated lipid metabolic responses in ischemia reperfusion injury

**DOI:** 10.1038/s41598-020-68995-z

**Published:** 2020-07-21

**Authors:** Ashim K. Bagchi, Arun Surendran, Akshi Malik, Davinder S. Jassal, Amir Ravandi, Pawan K. Singal

**Affiliations:** 1 Institute of Cardiovascular Sciences, St. Boniface Hospital Albrechtsen Research Centre, 351 Tache Ave. Room R3022, Winnipeg, MB R2H 2A6 Canada; 2 Department of Physiology and Pathophysiology, St. Boniface Hospital Albrechtsen Research Centre, 351 Tache Ave. Room R3022, Winnipeg, MB R2H 2A6 Canada; 3 Section of Cardiology, Max Rady College of Medicine, Rady Faculty of Health Sciences, University of Manitoba, Winnipeg, Canada

**Keywords:** Cardiovascular biology, Cardiovascular biology

## Abstract

Oxidized phospholipids (OxPLs) promote inflammation as well as low density lipoprotein (LDL) uptake in a variety of physiological and pathological states. Given the anti-inflammatory role of the cytokine IL-10, we investigated its modulatory effect on the production of oxidized phosphatidylcholines (OxPCs) as well as lipid metabolic responses in global myocardial ischemia/reperfusion (I/R) injury. Increased OxPCs levels, by 1-Palmitoyl-2-(5-oxovaleryl)-sn-glycero-3-phosphocholine (POVPC), promoted oxidative stress (OS) and cell death. OxPCs-mediated-OS, resulted in oxidized low-density lipoprotein receptor 1 (LOX-1) activation and upregulated the expression of toll-like receptor 2 (TLR2). IL-10-induced increase in proprotein convertase subtilisin/kexin type 9 (PCSK9) negatively regulated LOX-1 as well as TLR2 inflammatory responses. Under stress conditions, phosphorylation of sterol regulatory element binding protein 1c (SREBP 1c) was prevented by IL-10. The latter also prevented the generation of OxPCs and reduced their ratio (OxPCs/PCs) during injury. LOX-1 activation also promoted SREBP1c-mediated TGF-βRII expression which was inhibited by IL-10. Both fragmented and non-fragmented OxPCs were elevated during I/R and this effect was attenuated by IL-10. The largest impact (two–threefold change at log_2_) was on PAzPC, (1-palmitoyl-2-azelaoyl-sn-glycero-3-phosphocholine)—a fragmented OxPC. Thus it appears that among different OxPCs, IL-10 significantly reduces a single molecule (PAzPC)-mediated lipid metabolic responses in cardiomyocytes thereby mitigating inflammation and cell death.

## Introduction

One of the important biological phenomenon of all cardiac pathological conditions is increased oxidative stress (OS) through a major contribution by lipid peroxidation byproducts. Reactive oxygen species (ROS) cause oxidation of membrane phospholipids (OxPLs) and the formation of oxidative phosphatidylcholines (OxPCs)^1–3^. These OxPCs, particularly fragmented, are implicated in various inflammatory diseases including atherosclerosis and ischemia/reperfusion (I/R) injury in the heart^1,4^. It has been suggested that the inflammatory action of OxPLs is mediated by TLR2 but not through TLR4^5^. However, steps leading to these OxPCs effects on the receptor modifications are not known. In this regard, esterified PCs undergo enzymatic or non-enzymatic oxidation and form non-fragmented and fragmented OxPCs and perform different biological actions. Non-fragmented OxPCs induce adaptive responses through barrier-protective effects whereas fragmented OxPCs participate in the pathogenesis of diseases including atherosclerosis and other cardiovascular complications. Furthermore, one of the derivatives of the fragmented oxidized phospholipids, 1-palmitoyl-2-(5-oxovaleryl)-sn-glycero-3-phosphocholine (POVPC), is a known potent protein modifier due to a presence of the aldehyde group^6^. These OxPCs may bind covalently with free amino groups of different receptors and activate signaling pathways^7,8^. The role of these OxPCs in stress-induced cell signaling has been shown through the phosphorylation of p38 mitogen-activated protein kinase (p38MAPK) which is known to promote apoptosis^9–12^.

Lectin-like oxidized low density lioprotein (LDL) receptor-1 (LOX-1) is synthesized as a 40 kDa precursor protein with N-linked high mannose-type carbohydrate. Depending on the stimuli, it is glycosylated, processed and matured into a 50 kDa^13^. In general, LOX-1 is not efficiently transported to the cell surface but retained in the endoplasmic reticulum (ER) while matured (glycosylated) LOX-1 is transported to the cell surface. Furthermore, un-glycosylated LOX-1 has very low affinity for oxLDL binding^14^. While, mature LOX-1 supports the binding, internalization, and proteolytic degradation of OxLDL it does not support acetylation of LDL^15^. LOX-1 is known to induce TLR-2 activation and promote atherosclerosis^5^. During cardiac dysfunction, increased expression of LOX-1 has also been shown to stimulate superoxide radical formation through NADPH oxidase complex^16,17^. LOX-1 also increases the expression of TLR9 and initiates NF-κB-mediated immune response^13^. Proprotein convertase subtilisin/kexin type 9 (PCSK9) is an enzyme produced mainly in kidney and liver^18^. This protein plays a critical role in cholesterol homeostasis through its binding with LOX-1^19,20^. Furthermore, PCSK9 has been associated with the reduced risk of heart disease by lowering cholesterol levels^21,22^. Since blocking of cholesterol synthesis by 3-hydroxy-3-methylglutaryl CoA (HMG CoA) reductase blocked the expression of sterol regulatory element binding protein (SREBP1c) mRNA^23^, the cholesterol homeostasis may also be regulated by SREBP-1c. Studies also revealed that the SREBP1c mRNA expression was restored by cholesterol receptor ligand stimulations^24^.

There is strong evidence supporting the role of LOX-1 in pro-inflammatory response during atherosclerosis leading to heart failure^25^. However, there is no direct evidence whether these LOX-1 receptors participate in initiating cardiomyocyte–specific innate inflammatory response. In the setting of heart failure, post myocardial infarction (MI), we and others have shown that there is an upregulation of pro-inflammatory cytokine and tumor necrosis factor -α (TNF-α)^26,27^. Anti-inflammatory cytokine interleukin-10 (IL-10), mitigated deleterious effects of TNF-α^28–33^. Increased levels of OxPCs by exogenous TNF-α have been shown to promote OS and cell death in jurkat cells^34^. Studies also showed that IL-10 inhibited POVPC-induced cell–cell interaction as well as atherosclerotic lesion formation in in-vivo mice model^35^ by modulating LOX-1 receptor^22^. Concurrently, silencing of PCSK9 also reversed the OxLDL effects on LOX-1 receptors by inhibiting TNF-α, IL-1β and IL-6 production^36,37^. Recently, we have shown that inhibition of OxPCs activity by anti-OxPC antibody E06 caused a significant reduction in MI^4^.

We tested whether fragmented OxPCs activate LOX-1 and promote inflammatory processes by upregulating TLR2. We also examined the role of IL-10 in modulating the production of OxPCs during I/R injury and their effects on the myocardium.

## Results

### Effects of IL-10 on OxPCs levels and POVPC-induced OS

**Figure 1:**
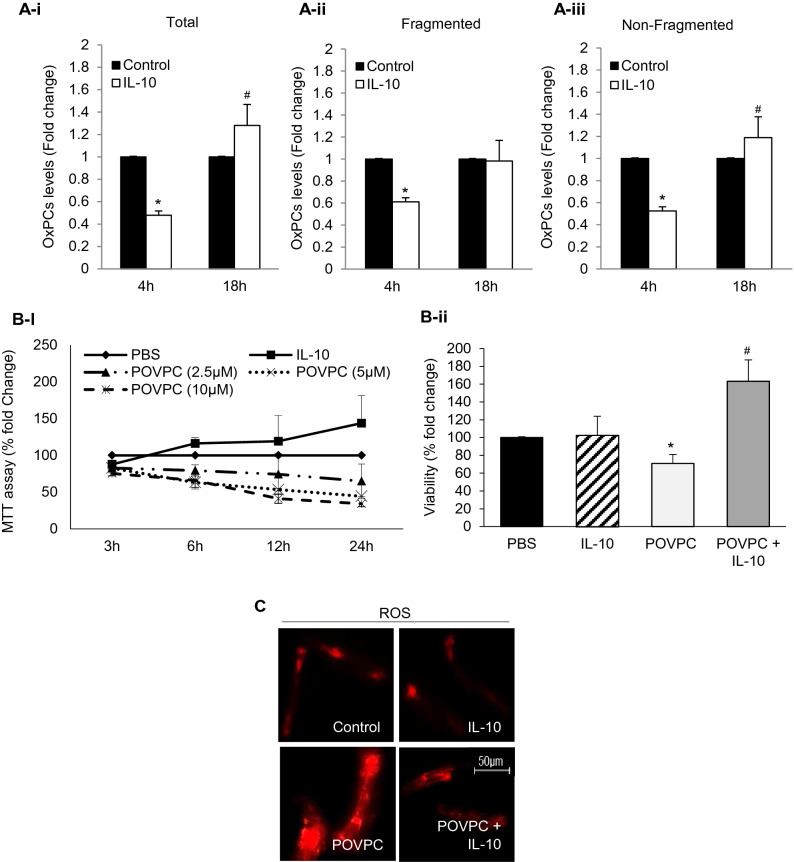
IL-10 prevents generation of OxPCs and improves cardiomyocyte viability during OxPCs-induced oxidative stress. (**A**) Total (**A-i**), fragmented (**A-ii**) and non-fragmented (**A-iii**) OxPCs were measured using mass-spectrometry at 4 h and 18 h post- IL-10 (10 ng/ml) stimulation. Data are represented as mean ± SE; *P < 0.01 and ^#^P < 0.05 vs. Control. (**B**) Percentage of viable cardiomyocytes was measured at different time points using 2.5–10 µM of POVPC (**B-i**). 5 µM of POVPC was considered as LC_50_ where cell viability was around 50% at 12 h. This concentration was used to treat cardiomyocytes 6 h prior to incubation with IL-10 (10 ng/ml) for 12 h (**B-ii**). Data are shown as mean ± SE. *P < 0.01 vs. Control, IL-10 alone. ^#^P < 0.05 vs. POVPC. (**C**) ROS was measured at 4 h post- IL-10 (10 ng/ml) treatment in cardiomyocytes that were pre-treated with POVPC (5 µM). Images are representative of three independent experiments showing magnified area with ROS generation using fluorescent dye coupled with Alexa 555 (Red). Magnification ×40.

Baseline production of total, fragmented and non-fragmented OxPCs was analyzed in isolated cardiomyocytes, with and without IL-10 for 4 h and 18 h (Fig. 1A-i,-ii, -iii) These data in the control cardiomyocytes for each fraction was normalized as 100% and compared with that in the presence of IL-10. At 4 h, the IL-10 caused a 40–50% reduction in OxPCs in each fraction and the change was significant (P < 0.01). However, at 18 h with IL-10, there was a rise (p < 0.05) in total OxPCs to 125% and this increase was mainly due to an increase in the non-fragmented group of OxPCs (Fig. 1A-iii). A dose-dependent (2.5–10 µM) and time-course (3–24 h) study was done after exposure to POVPC, to investigate the effect of IL-10 on OxPCs-induced cardiomyocyte cell viability (Fig. 1B-i). The cell viability was reduced by POVPC in a dose and time dependent manner. At an intermediate dose of 5 µM POVPC, cell viability at 6 h of incubation was noted to be > 60% (Fig. 1B-i) of the control. This dose and time were used for in vitro experiments to study the effect of IL-10 on OxPCs-mediated oxidative damage as well as cellular and molecular changes. IL-10 significantly improved viability (60%) of cardiomyocytes compared with control but was more than double compared to POVPC treatment alone (Fig. 1B-ii). While, non-oxidized OxPC compound PSPC, did not show any adverse effect on isolated cardiomyocytes rather there was a dose-dependent improvement in cell viability (Supplementary Fig. 1). Exposure to 5 µM POVPC caused a significant increase in ROS production which was significantly blocked by IL-10 (Fig. 1C).

### Effects of IL-10 on POVPC-induced LOX-1 and TLR2 expression

**Figure 2:**
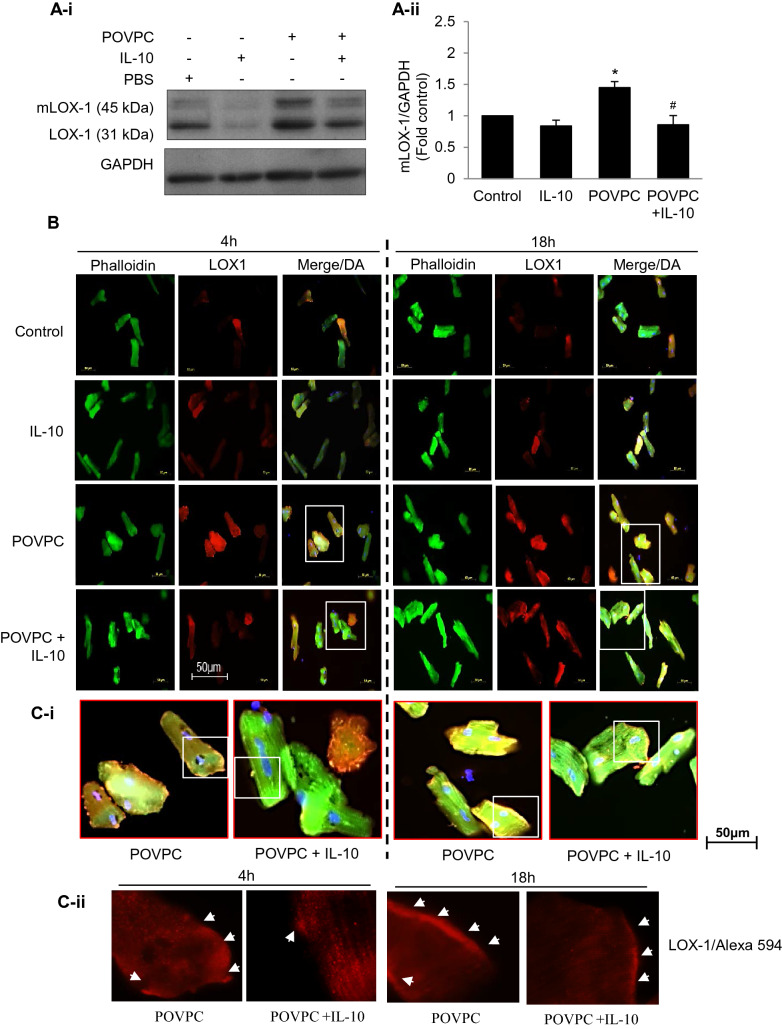
IL-10 prevents oxidized LDL receptor (LOX1) expression in POVPC-treated cardiomyocytes. (**A-i**) Western blots after 4 h of POPVC pre-treatment with or without IL-10 were done using specific LOX 1 antibody. GAPDH was used as a loading control. Representative of three independent experiments done for mature LOX1 (mLOX1). (**A-ii**) Histograms are shown as mean ± SE of N = 3 in duplicate. *P < 0.01 vs. respective controls, ^#^P < 0.05 vs. POVPC alone. (**B**) Adult cardiomyocytes pre-treated with 5 µM of POVPC were stimulated with or without IL-10 (10 ng/ml) for 4 and 18 h. Cells were fixed and incubated with specific LOX1 antibody and Phalloidin-Alexa 488 (Green) overnight, followed by secondary antibody labeled with Alexa 594 (Red). DAPI (Blue) was used to locate nuclei. Magnification = 40×. (**C-i**) White rectangles (from upper panels in **B**) were further enlarged to show positive cells for LOX1 expression. And white rectangles in **C-i** were enlarged in **C-ii** showing LOX-1 surface and subsurface localization.

POVPC-induced changes in the oxidized lipid associated LOX1 and mature LOX-1 (mLOX-1) in the presence or absence of IL-10 were studied (Fig. 2). There was a significant increase in both LOX-1 and mLOX-1 expression in POVPC treated cardiomyocytes at 4 h and IL-10 significantly blunted this upregulation (Fig. 2A-i and A-ii). In a quantitative analysis of mLOX-1, it was found to be upregulated 50% by POVPC and the change was mitigated by IL-10. Immunofluorescence studies at 4 and 18 h confirmed surface and subsurface localization of LOX-1 in cardiomyocytes treated with POVPC and that IL-10 markedly reduced this signal (Fig. 2B,C-i–ii—enlarged images from white boxes in 2C-i).

**Figure 3:**
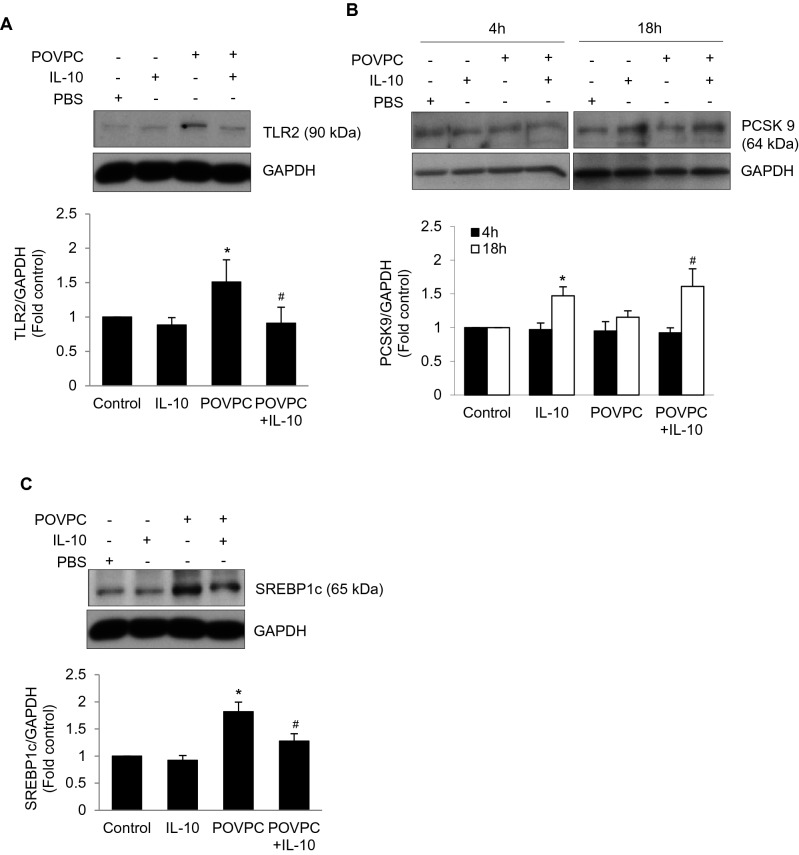
Cell lysates from 4 h of IL-10 treated cardiomyocytes were resolved on 8–12% PAGE gel followed by western blot using specific antibodies to TLR2, PCSK9, and SREBP1c^Ser372^ (**A**) Expression of TLR2. (**B**) Expression of PCSK9 and (**C**) Expression of SREBP1c^Ser372^. GAPDH was used as a loading control. Histograms are the representative of three independent experiments done in duplicate. Data are shown as mean ± SE of 3 experiments done in duplicate. *P < 0.01 Vs. Control, IL-10 and ^#^P < 0.05 vs. POVPC.

Since TLR2 expression is associated with cardiac injury and stress conditions^38^, we investigated the effect of POVPC on TLR2 expression which was increased at 4 h and was prevented by IL-10 (Fig. 3A). Since PCSK9 is known to cause LDL-receptor degradation^21^, this enzyme was studied under the condition of activation of LOX-1 and TLR2. We did not see any change in PCSK9 at 4 h. However, at 18 h, there was an increased PCSK9 response to IL-10 (Fig. 3B). In a supplementary study, we saw that inhibition of PCSK9 by PEP 2–8 (2 µM) promoted the LOX-1 activation in IL-10 treated cardiomyocytes (Supplementary Figure-2A). Further suggesting that IL-10 may have modulated LOX-1 expression via binding to PCSK9 and it was confirmed by immunoprecipitation of PCSK9 with LOX-1 expression (Supplementary Figure-2B). We did not see any significant expression of LOX-1 in PCSK9 inhibition (Supplementary Figure-2A). Nevertheless, IL-10 promoted binding of LOX-1 with PCSK9 (Supplementary Figure-2B). PCSK9 regulates LDL uptake via SREBP-1c and POVPC-induced increase in SREBP1c which was inhibited by IL-10 treatment (Fig. 3C).

### IL-10 effects on POVPC induced increase in Cyt c and troponin 1c

**Figure 4:**
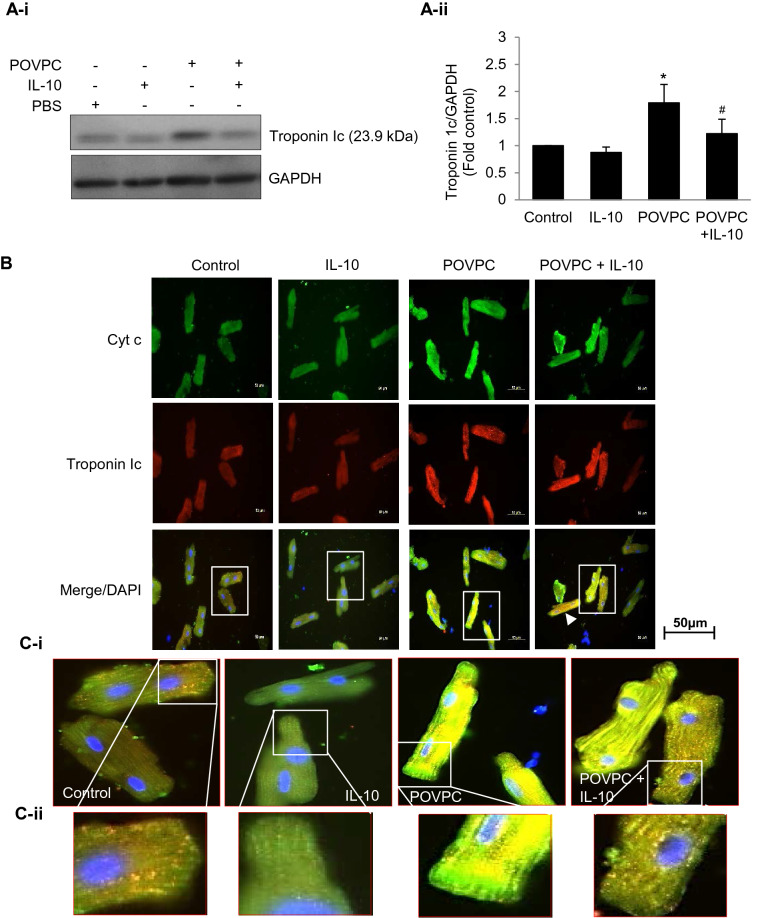
Co-localization of mitochondrial Cyt c and Troponin 1c. (**A**) Cardiomyocytes pre-treated with 5 µM of POVPC were stimulated with or without IL-10 (10 ng/ml) for 4 h. The cell lysates were analyzed by troponin 1c antibody (**A-i**). Histograms are the representative of three independent experiments done in duplicate (**A-ii**). Data are shown as mean ± SE. *P < 0.01 Vs. Control, IL-10 and ^#^P < 0.05 vs. POVPC. (**B**) Cells were fixed and incubated with specific CytC and Troponin 1c antibodies overnight followed by incubation with secondary antibody labeled with either Alexa 488 (Green) or Alexa 568 (Red). DAPI (Blue) was used to locate nuclei. Magnification = 40×. An increase in intensity for Cyt c and Troponin 1c was seen which was reduced by IL-10 treatment. (**C**) Photo enlargement of the areas in the correspondent rectangles shows fluorescence intensity (**C-i**) and an intracellular co-localization of Cyt c and Troponin 1c (**C-ii**).

LOX-1 is known to influence Cyt c and troponin 1c levels. Since POVPC upregulates LOX-1, we studied the effects of POVPC on troponin 1c and Cyt c. POVPC caused a significant (75%) increase in troponin 1c levels in cardiomyocytes and this effect was blunted by IL-10 but not completely (Fig. 4A-i–ii). For intracellular localization of troponin 1c, we used dual immunofluorescence approach for its co-localization with Cyt c in the mitochondria. POVPC treated cardiomyocytes showed profound increase in both troponin 1c and Cyt c which was mitigated by IL-10 (Fig. 4B). A mitochondrial localization of both was apparent at a higher magnification (Fig. 4C-i–ii).

### TLR2 innate signaling and OxPCs-mediated changes in I/R injury with IL-10

**Figure 5:**
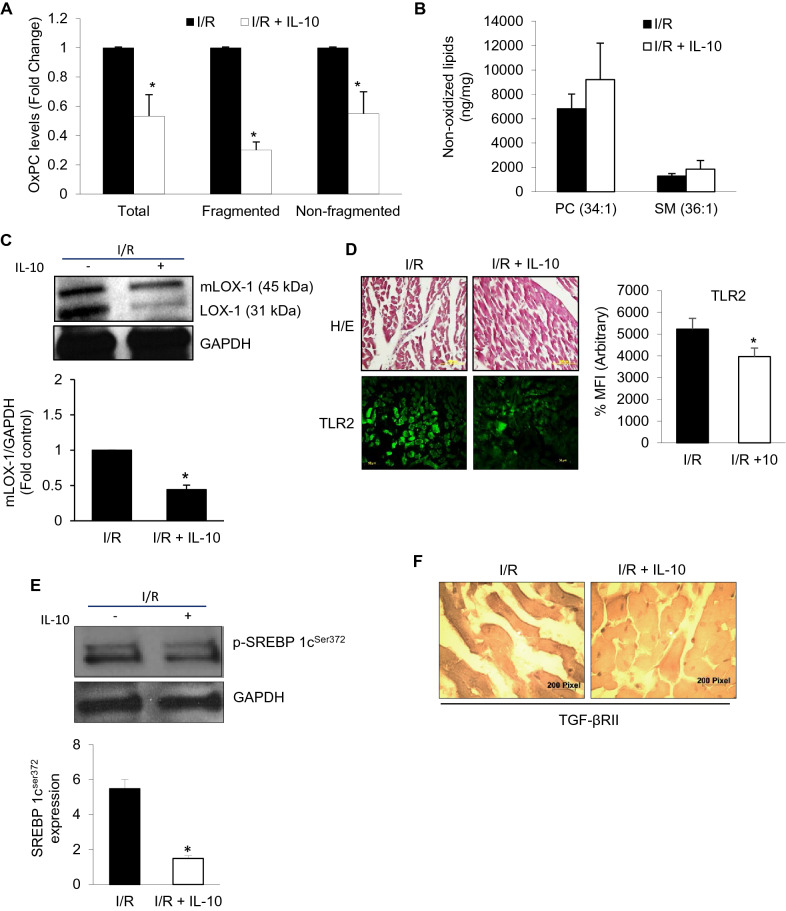
IL-10 modulates TLR2 innate signaling and OxPCs-mediated changes in global ischemia–reperfusion (I/R) injury. Hearts were subjected to global I/R with and without IL-10 as described in the methods. For IL-10 treatment, 10 ng/ml of IL-10 was added for 40 min during the reperfusion phase. (**A**) Total, fragmented and non-fragmented OxPCs were measured using mass-spectrometer for each fraction and their levels in I/R hearts were normalized compared with those in the presence of IL-10. (**B**) Non-oxidized lipids (*PC* Phosphatidylcholine and *SM* Sphingomyelin). (**C**) Western blotting was used to determine protein expression of mLOX1 and LOX1 post-I/R. (**D**) Expression of TLR2 was detected in the myocardium using immunofluorescence. Percentage of mean fluorescence intensity (% MFI) was calculated from three different heart sections and counted in 10 different fields after TLR2 staining. Upper panel is showing for Hematoxylin and Eosin (H/E) staining. (**E**) Western blots were probed with antibody for SREBP 1c^ser372^ . GAPDH was used as a loading control. Histogram (E, lower panel) is representative of 3 independent hearts probed for SREBP1c^ser372^. (**F**) TGF-β receptor II (TGF-βRII) expression was also studied in tissue sections using ABC peroxidase staining. Sections were probed with specific TGF-βRII antibody followed by diaminobezidine (DAB) staining.

Ex vivo global I/R with and without IL-10 was performed in adult rat hearts to follow changes in OxPCs (Total, fragmented and non-fragmented) levels (Fig. 5A). Myocardial levels of OxPCs under normal conditions are very nominal, however, global I/R caused an increase in the amount of total OxPCs which was reduced to 50% by IL-10 (Fig. 5A). This change was associated with a similar reduction in non-fragmented OxPCs while fragmented OxPCs were reduced to 30% by IL-10. However, we did not see any significant change in the non-oxidized PC (34:1) and SM (36:1) contents which were insignificantly higher than I/R groups alone (Fig. 5B). OxPCs associated lipid receptor, LOX-1 was found to be significantly higher in I/R and IL-10 reduced its receptor maturation (mLOX-1) as well as activation (LOX1) (Fig. 5C). In a quantitative analysis, IL-10 was found to reduce mLOX-1 by more than 50% (Fig. 5C, lower panel). Furthermore, I/R-induced increase in LOX-1 stimulated TLR2 expression which was mitigated by IL-10 (Fig. 5D). An increase in SREBP1c in I/R injury was also inhibited by IL-10 by more than threefold (Fig. 5E). Inflammation due to increase TLR2 was associated with TGF-β receptor II activation in I/R injured heart which was reduced by IL-10 (Fig. 5F).

### Differential patterns of OxPCs

**Figure 6:**
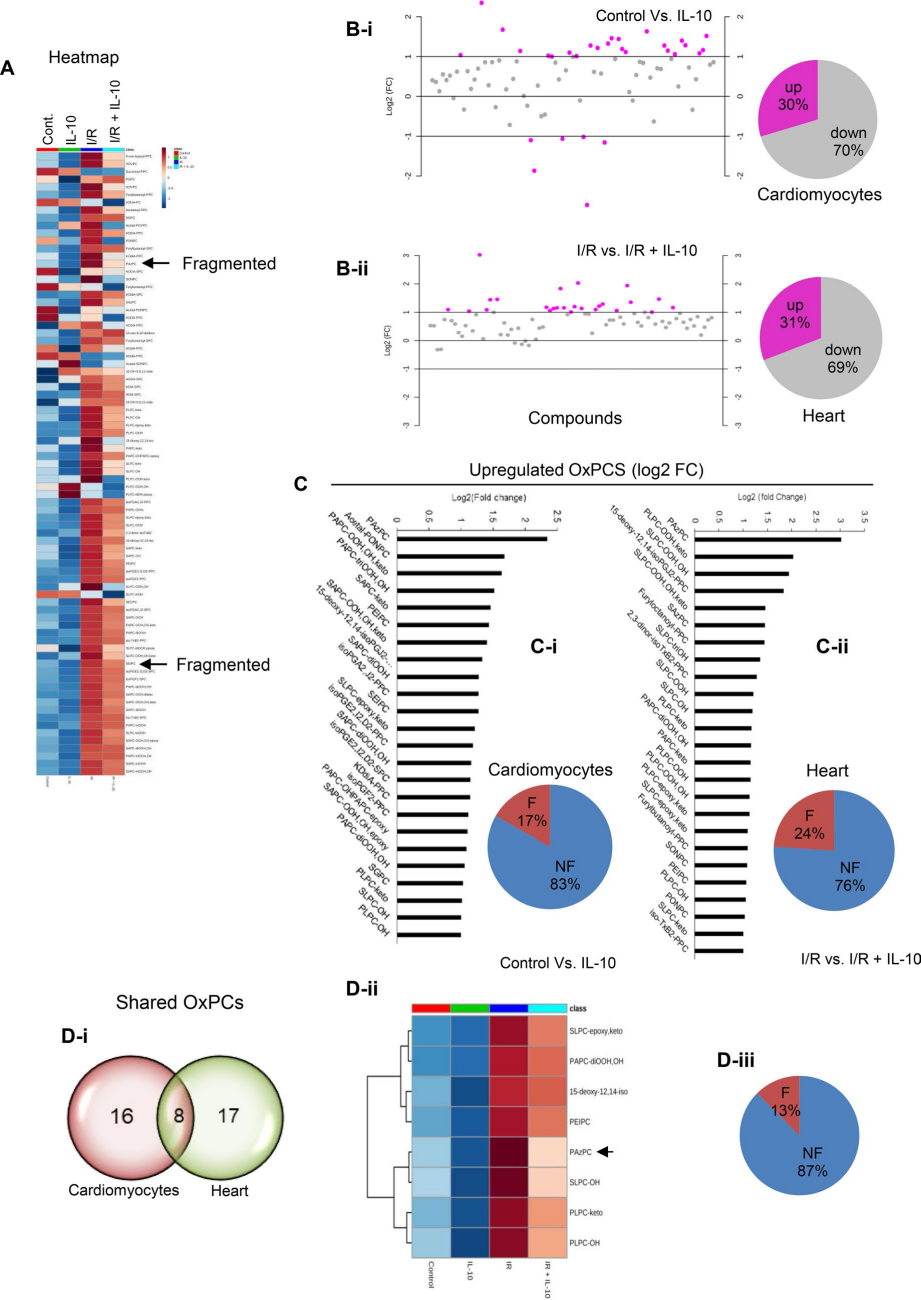
Differential changes in OxPCs levels in the cardiomyocytes and I/R heart after IL-10 treatment. (**A**) Heatmap was generated for all 80 oxidized phospholipids in pooled samples of cardiomyocytes (Control and IL-10 treated) and hearts (Global ischemia reperfusion (I/R) and I/R + IL-10) using mass-spectrometry. Color-bar on the top right, indicates concentration of OxPCs from high to low (Red through blue). (**B**) Scatter plot was generated to represent fold change (FC) at log2 after comparison of groups with or without IL-10 treatment. Pie diagrams represent percentage of OxPCs upregulated and/or downregulated in each condition (Cardiomyocytes; **B-i** and hearts; **B-ii**) after IL-10 treatment. (**C**) Bar diagrams represent fold-change of OxPCs levels higher than 1.0 at log 2. Pie diagram here depicts percentage of fragmented (F) and non-fragmented (NF) OxPCs levels in each condition (cardiomyocytes, **C-I**; and heart, **C-ii**) after IL-10 treatment. (**D**) Differentially expressed OxPCs in the stressed cardiomyocytes and heart at FDR < 0.001 using one-way Anova: (**D-i**) Venn diagram was generated to show shared OxPCs molecules between cardiomyocytes and heart tissue; (**D-ii**) Heatmap generated from commonly affected OxPCs by IL-10 and (**D-iii**) Percentage of these shared OxPCs showing F and NF molecules. Arrow in the heatmaps depicted most abundantly fragmented OxPCs during stress conditions underwent significant change by IL-10.

In order to evaluate specific OxPCs that were impacted by IL-10, we generated heatmaps for all eighty (80) fragmented and non-fragmented OxPCs. This was done to visualize the specific change in the concentration (from high to low represented by red through orange to blue) of OxPCs molecules across the two different conditions. Most fragmented OxPCs were significantly upregulated (orange to red) during I/R injury and were downregulated (orange, navy blue to dark blue) upon IL-10 treatment (Fig. 6A). On an average, about 30% of the total OxPCs were significantly (at log_2_ > 1.0 FC) reduced upon IL-10 treatment (Fig. 6B-i,-ii). Among these alterations in OxPCs molecules, 17% were fragmented compounds (PAzPC, Acetal-PONPC, KOdiA-PPC and SGPC) in cardiomyocytes (Fig. 6C-i; Supplementary Table 1) and 24% (PAzPC, SAzPC, Furyloctanoyl-PPC, Furylbutanoyl-PPC, SONPC, PONPC) were in I/R hearts (Fig. 6C-ii; Supplementary table 2). However, majority of these changes were due to a shift in non-fragmented OxPCs (Supplementary Tables 1 & 2), represented in bar graph (Fig. 6C). Our analysis of these OxPCs, found that PAzPC was the only compound which was maximally (2.5–3.0 fold at log_2_) modulated by IL-10 in cardiomyocyte as well as in the heart (Table 1; Fig. 6C-i-ii; bar diagrams). We also found a second major fragmented OxPC, SAzPC (1.46fold at log_2_) which was significantly reduced by IL-10 only in the heart but not in cardiomyocyte (Supplementary Table 2; Supplementary Fig. 3 and Arrows in Fig. 6A). Venn diagram depicted that there were 8 shared OxPCs which were commonly affected by IL-10 in cardiomyocytes and I/R hearts (Table 1; Fig. 6D-i). Heatmap of these 8 shared OxPCs (Fig. 6D-ii) showed changes due to IL-10 treatment. One out of these 8 shared OxPCs- PAzPC was dramatically modulated by IL-10 (Fig. 6D-iii). This fragmented OxPCs underwent a significant (P < 0.0001) shift in cardiomyocytes (light sky blue to Dark blue shown in Fig. 6D-ii) as well as in the heart (Red to light pink shown in Fig. 6D-ii) with IL-10 treatment as was evident in the heatmap (Fig D-ii; and Table 1; and Arrows in Fig. 6A).

**Table 1:** Common OxPCs compounds modulated by IL-10, both in cardiomyocytes and I/R heart.

Cardiomyocytes			
OxPCs compounds/number	F/NF	Fold change	log_2_(FC)
PAzPC/I	F	5.0928	2.3485
PEIPC/II	NF	2.7145	1.4407
15-deoxy-12,14-isoPGJ2-PPC/III	NF	2.5057	1.3252
SLPC-epoxy,keto/IV	NF	2.3247	1.217
PAPC-diOOH,OH/V	NF	2.0813	1.0575
PLPC-keto/VI	NF	2.0295	1.0211
SLPC-OH/VII	NF	2.0158	1.0114
PLPC-OH/VIII	NF	2.0004	1.0003

Heart			
OxPCs compounds/number	F/NF	Fold change	log_2_(FC)
PAzPC/I	F	8.1404	3.0251
15-deoxy-12,14-isoPGJ2-PPC/III	NF	3.5674	1.8349
SLPC-OH/VII	NF	2.2922	1.1968
PLPC-keto/VI	NF	2.2577	1.1748
PAPC-diOOH,OH/V	NF	2.2382	1.1623
SLPC-epoxy,keto/IV	NF	2.1379	1.0962
PEIPC/II	NF	2.0844	1.0596
PLPC-OH/VIII	NF	2.0788	1.0558

Fold change analysis at log_2_ > 1.0; *F* Fragmented, *NF* Non-Fragmented.

Numbers (I–VIII) have been arbitrarily assigned for a ready comparison between the cell and Heart OxPCs compounds.

## Discussion

We have previously reported that during global I/R injury, the recovery of the left ventricle performance was improved by the addition of IL-10 during reperfusion^38^. In this report, we show that IL-10 induced protection during I/R may be through an attenuation of fragmented OxPC activity. We have shown that the significant increase in fragmented OxPCs during I/R is inhibited by the addition of IL-10. It is known that these OxPCs have a significant role in I/R mediated myocardial injury^4^. Now, we show that increased OxPCs levels in I/R injured heart are associated with TLR2 expression which was prevented by IL-10. It demonstrates that TLR2-mediated inflammation is mediated by OxPCs, generated during I/R as well as in isolated cardiomyocytes and these observations are also supported by others^39,40^. Previous reports have shown that these oxidized phospholipids are the subsets of the oxidation-specific epitopes (OSE)^40^. These OSE are recognized as damage-associated molecular patterns (DAMPs) which tend to trigger inflammation^39,40^ by TLR2 in I/R. We have earlier reported that during I/R injury, there was an increase TNF-α receptor-associated changes in TRAIP and TRADD due to increased TLR2 activation^38^. This response was also associated with TGF-β receptor type I (TGF-βRI) and promoted fibrosis. Earlier, we have determined fibrosis by measuring TGF-β protein levels in IL-10 treated stressed cardiomyocytes and the heart^38^. In our global I/R model, level of fibrosis was not intense however, we have noticed some inhibition of the fibrotic process by IL-10 but this inhibition was not significant (data not shown). It is also known that TGF-β-mediated fibrosis signaling is mediated by SREBP1c and there is increased expression and nuclear translocation of SREBP1c in mice overexpressing TGF-β genes^41,42^. Thus SREBP1c may regulate pro-fibrotic effect of TGF-β via binding to its type II receptor (TGF-βRII) to activate and phosphorylate type I receptor (TGF-βRI) and regulate matrix protein synthesis^24^. Oxidation of phospholipids and production of OxPCs tend to promote LOX-1 in I/R injury which is largely expressed in cardiomyocytes. Maturation of LOX-1 during I/R injury was inhibited by IL-10 treatment with reduced SREBP1c synthesis. Our data demonstrates that fragmented OxPCs-induced LOX-1-mediated increase in TLR2 during myocardial global I/R injury and that this increase is associated with TGF-β receptor II activation which was inhibited by IL-10 treatment.

### IL-10 improves OxPC-mediated changes in cardiomyocyte

We have previously shown that exposure of isolated cardiomyocytes to fragmented phospholipids POVPC results in cell death^4^. We now show that the treatment of the cell with IL-10 can reduce OxPC-induced cell death by 50%. Mass spectrometric analysis of cardiomyocytes demonstrated a significant increase in fragmented oxidized phospholipids and improved survival due to IL-10 correlated with a significant reduction in overall OxPC levels. Furthermore, POVPC promoted LOX-1 expression in vivo as well as in vitro*.* It has been reported that LOX-1 interferes with lipid metabolism by promoting oxLDL uptake^43^. Increased LOX-1 expression in our studies was also associated with increased mitochondrial ROS production which was inhibited by IL-10. Increased ROS production by mitochondria in tissues has been reported by others^44,45^. Thus it is clear that IL-10 has the potential to reduce oxidized lipid-associated receptor activation during oxidation of phospholipids. Although PCSK9 expression in cardiomyocyte was unchanged at 4 and 18 h exposure to POVPC, its expression was significantly higher at 18 h of IL-10 pretreatment with or without POVPC. These data show that IL-10 maintained or improved PCSK9 levels and downregulated the increased LOX-1 expression due to I/R stress. IL-10 was able to overcome PCSK9 inhibition and promoted both PCSK9 and LOX-1 (Supplementary Fig. 2A,B) suggesting that there might be a feedback regulation of PCSK9 upon LOX-1 activation in the presence of IL-10. The PCSK9 inhibition studies also suggest that IL-10 negatively regulates LOX-1 expression (Supplementary Fig. 2A,B) and suppressed its activation during lipid oxidation by POVPC. Also, IL-10 mediated PCSK9 signals are required for LOX-1 inhibition.

We have shown that IL-10 blunted TNF-α-induced cell death and inflammatory response by modulating TLR2^38^. IL-10 is known to reduce the ROS production as well as cell death^29,31^ via Jak/Stat3 and AKT^28^. In the present study also, IL-10 mitigated cardiomyocyte death due to ROS generated by POVPC. Since phospholipid oxidation is reported to induce inflammatory response^46,47^ by triggering TLR2^5^, POVPC-induced OxPC and LOX-1 activation may have also elicited TLR2 activation to trigger inflammation which was mitigated by IL-10.

Increased expression of SREBP1c during phospholipid oxidation was inhibited by IL-10 suggesting that PCSK9 might differently regulate SREBP1c and control downstream LOX-1. It is known that SREBP1c plays a vital role in lipid synthesis as well as its uptake^21,48,49^ and thus its inhibition by IL-10 may be important to treat lipid metabolic diseases. Furthermore, our co-localization studies demonstrated that there was an increased troponin 1c together with Cyt c in POVPC treated cardiomyocytes suggesting that there is an increased mitochondrial stress during oxidation of phospholipids. Increased LOX-1 levels were associated with cardiac troponin 1c levels further indicating that LOX-1 may have promoted troponin 1c levels in the cardiomyocyte. We have shown previously that during cardiomyocyte I/R injury that there is specific enrichment of fragmented OxPC within mitochondria^4^ Apparently, IL-10 treatment ameliorated POVPC-induced mitochondrial stress by reducing Cyt c and troponin 1c expression in cardiomyocytes. Thus IL-10 treatment by reducing OxPCs-induced OS, lipid metabolic responses and TLR2 improves cardiomyocyte cell survival.

### Modulation of cardiomyocyte and heart-specific OxPCs by IL-10

During global I/R injury there was a significant increase in OxPCs (FDR < 0.001) and this was mainly associated with 6 (24%) fragmented OxPCs out of 25 species. Cardiomyocyte-specific modulation in these fragmented OxPCs by IL-10 was identified in that PAzPC, a homologue of 1-palmityl-2-(5-glutaryl)-sn-glycero-3-phosphocholine (PGPC) was maximally modulated by IL-10 in both conditions (in vitro and in vivo) and was significant (FDR < 0.0006). It appears that PAzPC induced cell death is related to phosphatidylserine (PS) oxidation causing release of mitochondrial Cyt c and promote apoptosis-inducing factor (AIF)^50^. Mitochondrial dysfunction and apoptosis due to OxPC is favorably influenced by hexadecyl azeloyl glycerophosphocoline, a homologue of PAzPC^48^ which was affected by IL-10 treatment suggesting that IL-10 modulates PAzPC and controls mitochondrial release of Cyt c and troponin 1c. Our previous work has shown that homologue of POVPC, PONPC results in mitochondrial dysfunction through a Bnip-3 mediated cell death pathway^4^. Protein–ligand molecular docking system depicted that LPAzPC, an inhibitor of PAzPC directly interacts and binds with LOX1 at Gln193 and Ser198 in one subunit and Ser160 in the other^51^ and blocks POVPC treated PAzPC-induced LOX-1 activation. PAzPC promoted LOX-1 activation in cardiomyocytes in the heart which may subsequently change lipid metabolic responses. And this whole chain was inhibited by IL-10.

In addition to cardiomyocyte-specific change in PAzPC by IL-10, another fragmented OxPC, SAzPC, a homologue of 1-stearoyl-2-glutaroyl-snglycero-3-phosphocholine (SGPC) was noted as a second major fragmented OxPCs compound also modulated by IL-10 but only in the heart. Since SAzPC levels were increased only in I/R hearts and not in isolated cardiomyocytes exposed to POVPC, it may be an indication of non-cardiomyocyte specific heart-response during I/R injury which may promote TGF-β-mediated fibrosis. Previous observations showed that SAzPC helps monocyte and endothelial cells to induce platelet activation causing thrombosis^52^.

**Figure 7:**
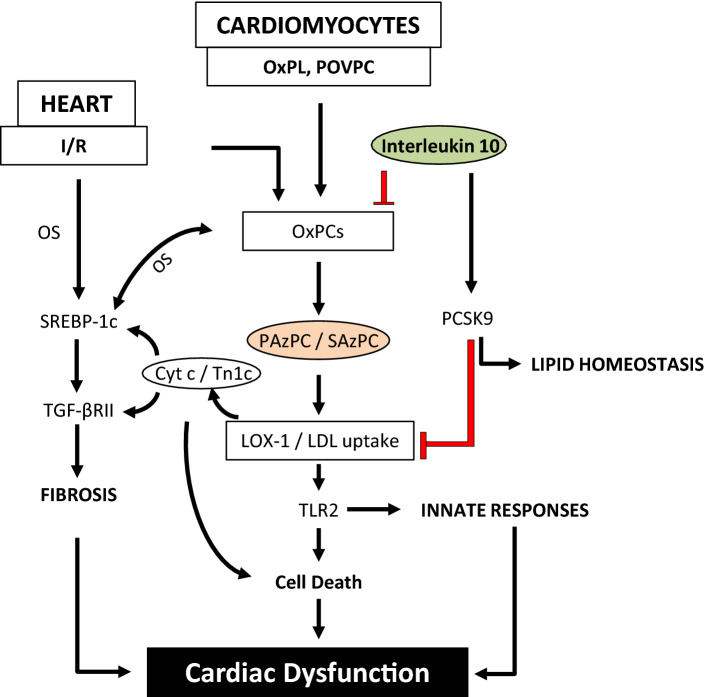
Schematic representation of the effects of IL-10 on OxPCs-induced OS-mediated lipid metabolic responses and cardiac dysfunction under stress conditions. Oxidation of phospholipids generates OxPCs and POVPC. Prolonged exposure to these stresses leads POVPC to further synthesize PAzPC and SAzPC, fragmented OxPCs compounds which upregulate oxidized LDL receptor (LOX-1). In vitro experiments using isolated adult rat cardiomyocytes treated with POVPC promoted oxidative stress (OS) in mutually supported manner. On the other hand, ex-vivo ischemia/reperfusion (I/R) experiments also suggest an increase in OxPCs along with alterations in lipid metabolic responses. Data suggest that increased PAzPC in the isolated cardiomyocytes or in I/R hearts may have interacted with LOX-1 to trigger cardiomyocyte-specific lipid metabolic responses and promoted lipid uptake. Co-localization study of troponin 1c and Cyt c suggest that increased SREBP1c activation is associated with their increase. It is known that SREBP 1c regulates TGF-β signaling pathway. We have earlier shown that I/R promoted TGF-β receptor II activation^38^ which in this study appears to be upregulated through LOX-1 mediated SREBP 1c activation. On the other hand, LOX-1 activation by PAzPC and/or SAzPC may promote inflammation/innate responses through TLR2 activation. Role of PCSK9 is largely known to regulate lipid homeostasis. Increased PCSK9 by IL-10 may prevent LOX-1 induced changes in metabolic and inflammatory responses. Both PAzPC and SAzPC were greatly reduced by IL-10. Heatmap studies indicated PAzPC to be cardiomyocyte specific in isolated cell study as well as in whole heart whereas, SAzPC, also a fragmented OxPC is a non-cardiomyocyte specific response. SAzPC was also significantly modulated by IL-10 in the I/R injured heart suggesting that SAzPC may provide a cumulative effect together with PAzPC during injury promoted lipid uptake, inflammation, cell death and IL-10 prevents these deleterious effects.

Enhanced OxPLs by OS as well as POVPC in cardiomyocyte and other cells in the heart resulted in an increased multiple derivatives of OxPCs including PAzPC and SAzPC (Fig. 7). PAzPC was increased by POVPC in cardiomyocytes as well as in I/R injury in the heart suggesting this change to be cardiomyocyte specific. Among 6 fragmented OxPCs in I/R heart, SAzPC was also significantly mitigated (1.4528 fold change at log_2_) by IL-10 but only in the heart. These two species appear to be potential protein signal transduction modifiers of many innate and adaptive immune responses in the heart. Stress-induced upregulation of oxidized LDL receptors (LOX-1) and production of mitochondrial Tn1c and Cyt c appear to be due to further downstream effects of PAzPC and SAzPC (Fig. 7). Occurrence of fibrosis through the upregulation of SREBP-1c and TGF-βRII as well as cell death through the upregulation of innate response and TLR2 ultimately results in cardiac dysfunction. PAzPC led to cardiomyocyte-specific damage perhaps by targeting proteins involved in cell cycle such as proliferating cell nuclear antigen (PCNA) and cyclin D as reported earlier in vascular smooth muscle cells^10^. Here, we also propose that PAzPC, may play the role of a “danger species” recognized by TLR2 present on cardiomyocyte and trigger OS-induced inflammation-mediated cardiac cell death. It has already been reported that a prolonged oxidation of POVPC give rise to PGPC and further PAzPC as an irreversible end-product^10^ which promotes TLR-2. Anti-inflammatory role of IL-10 may have reduced prolonged oxidation of phospholipids in cardiomyocytes and inhibited PAzPC synthesis and SAzPC in other cells of the heart. Thus cumulative effect of these two major species (PAzPC and SAzPC) in I/R injury condition may have worsen the OS response-mediated inflammation and cardiac cell death leading to heart damage which was mitigated by IL-10.

## Materials and methods

### Synthetic Standards and experimental protocols

1, 2-dinonanoyl-sn-glycero-3-phosphocholine (DNPC), 1-palmitoyl-2-(5-oxovaleryl)-sn-glycero-3-phosphocholine (POVPC), 1-palmitoyl-2-glutaroyl-sn-glycero-3-phosphocholine (PGPC), 1-stearoyl-2-glutaroyl-snglycero-3-phosphocholine (SGPC), 1-palmitoyl-2-azelaoyl-sn-glycero-3-phosphocholine (PAzPC), 1-palmitoyl-2-(9′-oxo-nonanoyl)-sn-glycero-3-phosphocholine (PONPC) and 1-stearoyl-2-azelaoyl-sn-glycero-3-phosphocholine (SAzPC) were obtained from Avanti Polar Lipids (Alabaster, AL). 1-(Palmitoyl)-2-(5-keto-6-octene-dioyl) phosphatidylcholine (KOdiA-PC) and 1-(palmitoyl)-2-(4-keto-dodec-3-ene-dioyl) phosphatidylcholine (KDdiA-PC) were purchased from Cayman Chemicals (Ann Arbor, MI). All solvents were HPLC grade. Animals were housed and maintained on regular chow and the methods and experimental protocols were approved by the University of Manitoba Animal Care Committee (Winnipeg, Manitoba, Canada) following the guidelines established by Canadian Council of Animal Care.

### Isolation of cardiomyocytes and treatment

A standard Langendorff perfusion method was used to isolate cardiomyocytes from normal adult male Sprague Dawley rat hearts (200–250 g)^26,53^. Briefly, isolated hearts were initially perfused with calcium free Krebs–Henseleit (KH) buffer (110 mM NaCl, 2.6 mM KCl, 1.2 mM KH2PO4, 1.2 mM MgSO4, 25 mM NaHCO3, 20 mM taurine, 11 mM glucose, pH 7.4) at 37 °C followed by the buffer containing 0.1% collagenase, 0.1% BSA, 50 μM CaCl2. After 40 min of perfusion, ventricles were removed and incubated in desegregation solution containing 1% BSA and 50 μM CaCl2. Homogenous suspension of cardiomyocytes was incubated overnight in Phenol red free-medium 199 (M199) supplemented with 10% fetal bovine serum (FBS) and 100U of pen/strep antibiotics. The viable cardiomyocytes were used in further studies. These cardiomyocytes were exposed to oxidized phospholipid POVPC (5 µM) for 4 h, prior to IL-10 (10 ng/ml) stimulation for 4 h and 18 h^52^. We used different concentrations of POVPC (2.5–10 µM) for different time points (3–24 h) for selecting the above mentioned duration as well as concentration. Phosphate buffer saline (PBS) was used as control. Apart from a fragmented OxPC compound, we have also used PSPC a non-oxidized OxPC compound at different dosages (2.5–10 µM) for different time points (3–24 h).

### Global I/R injury

Ex-vivo global ischemia/reperfusion (I/R) was performed using previously described method^38^. Briefly, adult rat heart aorta was cannulated and retro-perfusion was done with KH buffer in presence of 95% O_2_ and 5% CO_2_ at the rate of 8–10 ml/g/min at 37 °C. In each experiment, hearts were allowed to equilibrate for 10 min with the KH buffer perfusion containing 50 µM CaCl_2_ and were then subjected to global ischemia for 20 min followed by reperfusion for 90 min. Separately, hearts were also re-perfused for 40 min with IL-10 (10 ng/ml/g), followed by a wash out period of 50 min of re-perfusion with buffer only^38^. These timings for perfusion have been based on our previous study^38^.

### Cell viability and ROS measurement

To assess cell viability, treated or control cardiomyocytes (2 × 10^4^) on 96-well plates were incubated at 37 °C for 3 h with 5 mg/ml of MTT [3-(4,5-dimethylthiazol-2-yl)-2,5-diphenyltetrazolium bromide]^54^. After 3 h, supernatant was removed and 150 µl of MTT solvent (4 mM HCl, 0.1% NP40 in isopropanol) was added to each well. Plates were kept on an orbital shaker for 20 min. The MTT formazan was fully dissolved by pipetting few times and optical density was read at 590 nm using a plate reader (Dynex Technologies, Chantilly VA). Percentage of viable cells was counted using a standard formula: (OD_sample_—OD_Blank_)/(OD_control_—OD_Blank_) × 100. Reactive oxigen species, superoxide anion were measured in cardiomyocytes using 5 µM of dihydrooxyethidium (DHE). Cells were incubated for 30 min at 37 °C. Cells without DHE were considered as negative control. Images were captured under fluorescence microscopy using Olympus microscope (1 × 81) (Olympus AmericaInc. Melville, NY, USA).

### Western blotting

Cell or tissue lysates were used to run SDS-PAGE and 20–30 µg of proteins separated from the gel were transferred onto PVDF membrane for 1 h and 30 min at 200 mA at 4 °C. PVDF membranes were probed overnight using specific antibodies PCSK9 (Catalogue # 95478) and LOX-1 (Catalogue # 60178) (Abcam and Cell Signaling Technology, USA) after blocking with 5% fat free skimmed milk for 1 h at room temperature (RT). The membranes were further incubated with appropriate horseradish peroxidase (HRP)-conjugated secondary antibodies for 1 h, after proper washing (TBS-T with 1% skimmed milk), bands were visualized using ECL kit^38,53^.

### Histochemical and immunofluorescence studies

#### Adherent cardiomyocytes

As described before^53^, adhered cardiomyocytes were fixed in 4% paraformaldehyde and washed with PBS-Tween 20 (PBS-T) twice and incubated with 0.1% sodium citrate and 0.05% triton-X100 for 10 min at RT. Non-specific sites were blocked using 3% normal horse serum (NHS) for 1 h and washed twice with PBS-T. Specific primary antibodies were used to detect LOX-1, troponin 1c and Cyt c with Alexa 488, 568 or 594 conjugated secondary antibodies (Invitrogen, USA). Expression and co-localization of these antibodies in cardiomyocytes were analyzed and quantified by microscopic analysis (Carl Zeiss., LSM 5 Pa, Germany) attached with Zeiss AxioCam HRM camera HAL 100 lamp, using AxioVision 4.8 software.

#### Cardiac tissue sections

Tissue pieces from hearts after I/R with or without IL-10 were fixed in 4% paraformaldehyde and embedded in paraffin. Tissue Sections (5–6 µm) were mounted on microscopic slides and de-paraffinized in xylene and hydrated through graded series of alcohol. Sections were washed with PBS twice and permeabilized in 0.05% triton-X100 for 15 min at RT. Tissue endogenous peroxidase was inhibited by antigen retrieval process using enzymatic retrieval buffer (10 mM Tris base, 1 mM EDTA, 10 mM sodium citrate and 0.05% Tween 20, pH 9.0). Fc receptor sites were blocked using 3% NHS for 1 h and washed twice with PBS-T. Specific primary antibody was used to detect TLR2 with Alexa 488 conjugated secondary antibody (Invitrogen, USA). Antibody to TGF-βRII was also used and stained with diaminobenzidine (DAB) followed by Hematoxylin/Eosin staining^38^. Expression of these antibodies in cardiomyocytes was analyzed and quantified by microscopic analysis (Carl Zeiss., LSM 5 Pa, Germany) attached with Zeiss AxioCam HRM camera HAL 100 lamp, using AxioVision 4.8 software.

### Mass spectrometry analysis

#### Sample preparation

Phospholipids were extracted from differently treated cardiomyocytes and analyzed as previously described^4^. Briefly, cells were washed with PBS and kept under Nitrogen (N_2_) gas in 500 µl of methanol/acetic acid (3% v/v) solution containing 0.01% butylated hydroxytoluene (BHT). Each sample was mixed with five internal standards for quantitation^4^. Under the same N_2_ gas condition, these samples were vortexed and washed three times with hexane/BHT by centrifugation at 3500 rpm for 5 min at 4 °C. After each wash, hexane part from the mixture was removed carefully. Finally, 2 mL of chloroform/BHT and 750 µL of PBS were added to each tube and centrifuged. The lower organic layer was carefully removed and transferred in a new glass tube and dried using nitrogen evaporator. The lipid extracts were then reconstituted into 200 µL of chloroform/methanol (2:1v/v) for storage at − 80 °C.

#### High performance liquid chromatography (HPLC)

The separation of OxPLs was carried out using reverse-phase (RP) chromatography as reported previously^11^. Immediately prior to injection, extracts from hearts or cardiomyocyte were reconstituted in RP elute consisting of 60:40 acetonitrile:water, 10 mM ammonium formate and 0.1% formic acid. Thirty microliters of the sample were injected into an Ascentis Express C18 HPLC column (15 cm × 2.1 mm, 2.7 µm; Supelco Analytical, Bellefonte, Pennsylvania, USA) with separation by a Prominence UFLC system from Shimadzu Corporation (Canby, Oregon, USA). Elution was performed using a linear gradient of solvent A (acetonitrile/water, 60:40v/v) and solvent B (isopropanol/acetonitrile, 90:10v/v) with both solvents containing 10 mM ammonium formate and 0.1% formic acid. The mobile phase composition used was as follows: initial solvent B at 32% until 4 min; switched to 45% B; 5 min 52% B; 8 min 58% B; 11 min 66% B; 14 min 70% B; 18 min 75% B; 21 min 97% B; 25 min 97% B; 25.10 min 32% B. A flow rate of 260µL/min was used for analysis, and the sample tray and column oven were held at 4 and 45 °C, respectively.

Detection of OxPL was carried out by mass spectrometry in positive polarity mode. MRM scans were performed on 84 transients using a product ion of 184.3 m/z, corresponding to the cleaved phosphocholine moiety. The mass spectrometry setting were as follows: curtain gas, 26psi; collision gas, medium; ion spray voltage 5500 V; temperature 500 °C; ion source gas 1, 40psi; ion source gas 2, 30psi; declustering potential, 125 V, entrance potential, 10v; collision energy, 53 V; collision cell exit potential, 9 V; and dwell time, 50msec. A 4,000 QTRAP^®^ triple quadruple mass spectrometer system with a Turbo V electrospray ion source from AB Sciex (Framingham, Massachusetts, USA) was coupled to the liquid chromatography system.

### Statistical analysis

The statistical analysis was done using MetaboAnalyst v 4.0 software^55^. The data was first normalized using log-transformation and auto-scaling before performing the univariate analysis. The volcano plot, which is a combination of both fold change and t-tests, was used to identify the significant OxPC molecules compared to controls. In the volcano plot, fold change threshold (x) was set at 2.0 and t-tests threshold (y) was set at 0.1^56^. The threshold values were considered significant depending upon the position from the integers (0, 0). A heatmap was then constructed using the mean value of replicate runs to visualize the change in the concentration of OxPC molecules value across different conditions using dataset.

Densitometry analysis of immunoblots were done using Quantity One software. ImageJ software was used to measure fluorescence intensity^57^. Mean ± SE were calculated and One-way ANOVA followed by bonferroni’s test was applied. Data were considered to be significant at P < 0.05.

## Supplementary information

Supplementary file1
