# Supplementary material for: IL-10 attenuates OxPCs-mediated lipid metabolic responses in ischemia reperfusion injury
Source: Sci Rep. 2020 Jul 21;10:12120. doi: 10.1038/s41598-020-68995-z (PMC7374703; doi:10.1038/s41598-020-68995-z)
Supplement: Supplementary file 1 — Supplementary file1 [file 41598_2020_68995_MOESM1_ESM.pdf]

AMENDED SUPPLEMENTARY INFORMATION

(Ms # SREP-19-21227-T)

(*With Unprocessed original Full-Length Gels and Blots*)

IL-10 Attenuates OxPCs-Mediated Lipid Metabolic Responses in Ischemia Reperfusion Injury

Ashim K. Bagchi[1,2], Arun Surendran[1,2], Akshi Malik[1,2], Davinder S. Jassal[1,3], Amir Ravandi[1,3] and Pawan K. Singal[*1,2]

[1]Institute of Cardiovascular Sciences, St. Boniface Hospital Albrechtsen Research Centre, [2]Department of physiology and Pathophysiology; [3]Section of Cardiology, Max Rady College of Medicine, Rady Faculty of Health Sciences, University of Manitoba, Winnipeg, Canada.

Running Title: IL-10 in OxPCs-mediated lipid metabolic responses

[*]Corresponding Author:

Dr. Pawan K. Singal
Institute of Cardiovascular Sciences
St. Boniface Hospital Albrechtsen Research Centre
351 Tache Ave. Room R3022
Winnipeg, Manitoba, R2H 2A6. Canada
Tel: 204-235-3416; Fax: 204-233-6723
Email: psingal@sbrc.ca

Supplementary Materials and Methods:

Non-oxidized PSPC treatment: Isolated adult cardiomyocytes were treated with different dosages of PSPC (2.5 - 10µM) for different time points (3 – 24h). We observed, cardiomyocyte cell death due to PSPC using MTT cell viability assay as described in the material and methods section in the manuscript.

PCSK9 inhibition study: A standard dose of PEP 2-8, a PCSK9 inhibitor was used as per described by Zhang et. al., (2014). Isolated adult cardiomyocytes were pre-treated with 2µM of PEP 2-8 followed by IL-10 treatment for 4h and/or 18h under same culture conditions.

Immunoprecipitation: A previously described immunoprecipitation method was used with minor modification (Bagchi et al., 2013). Cell lysates were pre-cleared with 5µl of normal horse serum and 30µl of protein A/G-conjugated Sepharose beads for 1h at 4°C. Pre-cleared cell lysates were separated from beads by centrifugation at 7000g for 5min at 4°C and further incubated for overnight at 4°C with 2.5µl of appropriate PCSK9 antibodies and 100µl of the gel beads. Following day, Antigen-antibody complex bound to Protein A/G beads were collected by centrifugation at 12000 g for 15 min at 4°C. These complexes were washed three times in lysis buffer and eluted in 30µl of SDS-sample buffer by boiling for 5min. The samples (5µl) were resolved on 8% SDS-PAGE and probed with LOX-1 antibody by western blotting.

Brief methodology of western blot processing:

Set 1: Samples were run on 12% resolving gel and transfer onto PVDF membranes. These membranes were cut in two halves along the 50kDa protein standard marker. **Upper half/blot** (containing 200 – 50kDa protein bands) probed for PCSK9 rabbit antibody (MW = 64kDa) shown in Figure 3B and **Lower half/blot** (containing 50 – 20kDa protein bands) probed for Lox-1 Rabbit (MW = 30 - 45kDa) shown in Figure 2A and finally, only **Lower half/blot** was processed for GAPDH (37.5kDa) shown in both Figure 2A and Figure 3B.

Set 2: Samples were run on 8% resolving gel and transfer onto PVDF membranes. We cut these membranes in two halves along the 75kDA. **Upper half/blot** (containing 200 – 80kDa protein bands) for TLR2 mouse antibody (MW = 90kDa) shown in Figure 3A and **Lower half/blot** (containing 70 – 20kDa protein bands) probed for SREBP-1 Rabbit (MW = 65kDa) shown in Figure 3C. Finally, only **Lower half/blot** was processed for GAPDH (37.5kDa) shown in Figure 3A and 3C.

Set 3: Some of the whole/cut blots were re-probed with different antibodies from different sources. For example, those blot used for TLR2 (90kDa)/SREBP-1c (65kDa) antibodies were re-probed with Troponin (23.9kDa). Thus their GAPDH is correspondent to same blot as for samples on other half described in set 2.

References:

Bagchi, A. K., Sharma, A. K., Dhingra, S., Ludke, A. R. L., Al-Shudiefat, A. A. & Singal, P. K. Interleukin-10 activates Toll-like receptor 4 and requires MyD88 for cardiomyocyte survival. Cytokine 61, 304–314, doi: 10.1016/j.cyto.2012.10.013, (2013).

Zhang, Y. et.al. Identification of a Small Peptide That Inhibits PCSK9 Protein Binding to the Low Density Lipoprotein Receptor. J. Biol. Chem. 289, 942–955, doi: 10.1074/jbc.M113.514067 (2014).

***Supplementary Table 1**:* IL-10 modulated OxPC compounds in cardiomyocytes

OxPCs Compounds	F/NF	Fold Change	log_2(FC)
PAzPC	F	5.0928	2.3485
Acetal-PONPC	F	3.1956	1.6761
PAPC-OOH,OH,keto	NF	3.1004	1.6324
PAPC-triOOH,OH	NF	2.8644	1.5182
SAPC-keto	NF	2.7515	1.4602
PEIPC	NF	2.7145	1.4407
SAPC-OOH,OH,keto	NF	2.6400	1.4005
15-deoxy-12,14-isoPGJ2-PPC	NF	2.5057	1.3252
SAPC-diOOH	NF	2.4339	1.2832
isoPGA2,J2-PPC	NF	2.4225	1.2765
SEIPC	NF	2.4219	1.2762
SLPC-epoxy,keto	NF	2.3247	1.2170
isoPGE2,I2,D2-PPC	NF	2.2822	1.1904
SAPC-diOOH,OH	NF	2.2387	1.1627
isoPGE2,I2,D2-SPC	NF	2.2173	1.1488
KDdiA-PPC	F	2.2050	1.1408
isoPGF2-PPC	NF	2.1658	1.1149
PAPC-OHPAPC-epoxy	NF	2.1426	1.0993
SAPC-OOH,OH,epoxy	NF	2.1164	1.0816
PAPC-diOOH,OH	NF	2.0813	1.0575
SGPC	F	2.0516	1.0368
PLPC-keto	NF	2.0295	1.0211
SLPC-OH	NF	2.0158	1.0114
PLPC-OH	NF	2.0004	1.0003

Fold Change analysis at Log_2 >1.0; F = Fragmented, NF = Non-Fragmented

Supplementary Table 2: IL-10 modulated OxPC compounds in I/R heart

OxPCs Compounds	F/NF	Fold Change	log_2(FC)
PAzPC	F	8.1404	3.0251
PLPC-OOH,keto	NF	4.0871	2.0311
SLPC-OOH,OH	NF	3.8412	1.9415
15-deoxy-12,14-isoPGJ2-PPC	NF	3.5674	1.8349
SLPC-OOH,OH,keto	NF	2.7566	1.4629
SAzPC	F	2.7374	1.4528
Furyloctanoyl-PPC	F	2.7156	1.4413
SLPC-triOH	NF	2.5547	1.3532
2,3-dinor-isoTxB2-PPC	NF	2.4395	1.2866
SLPC-OOH	NF	2.3239	1.2165
SLPC-OH	NF	2.2922	1.1968
PLPC-keto	NF	2.2577	1.1748
PAPC-diOOH,OH	NF	2.2382	1.1623
PAPC-keto	NF	2.2278	1.1556
PLPC-OOH	NF	2.226	1.1545
PLPC-OOH,OH	NF	2.1967	1.1354
PLPC-epoxy,keto	NF	2.1873	1.1292
SLPC-epoxy,keto	NF	2.1379	1.0962
Furylbutanoyl-PPC	F	2.1317	1.092
SONPC	F	2.1223	1.0857
PEIPC	NF	2.0844	1.0596
PLPC-OH	NF	2.0788	1.0558
PONPC	F	2.0532	1.0379
SLPC-keto	NF	2.0112	1.0081
iso-TxB2-PPC	NF	2.0051	1.0037

Fold Change analysis at $Log_2 > 1.0$; F = Fragmented, NF = Non-Fragmented

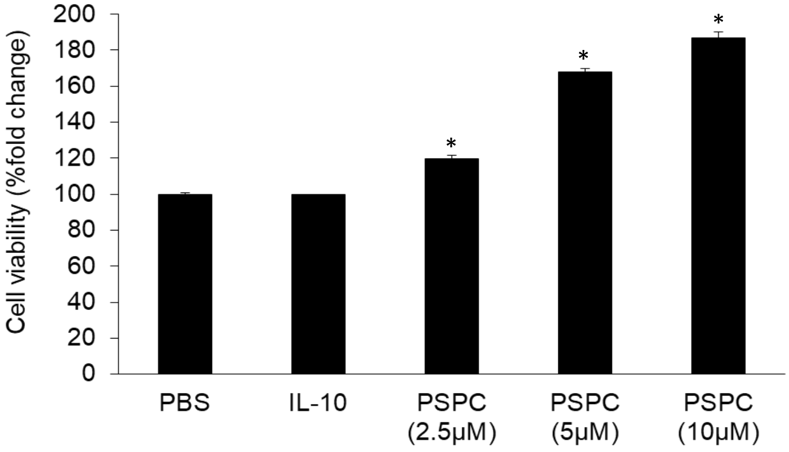

Supplementary Fig 1: Effect of PSPC (2.5 - 10µM), a non-fragmented and non-oxidized OxPCs compound on cardiomyocyte viability. Percentage of viable cardiomyocytes (in fold change) was measured at 24h of exposure with PSPC. Data are mean ± SE of three independent experiments. * $P < 0.01$ vs. Control or IL-10 alone.

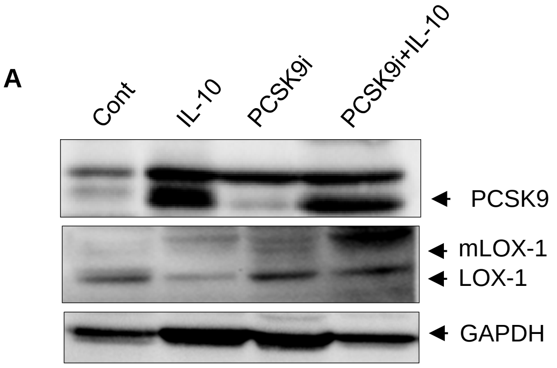

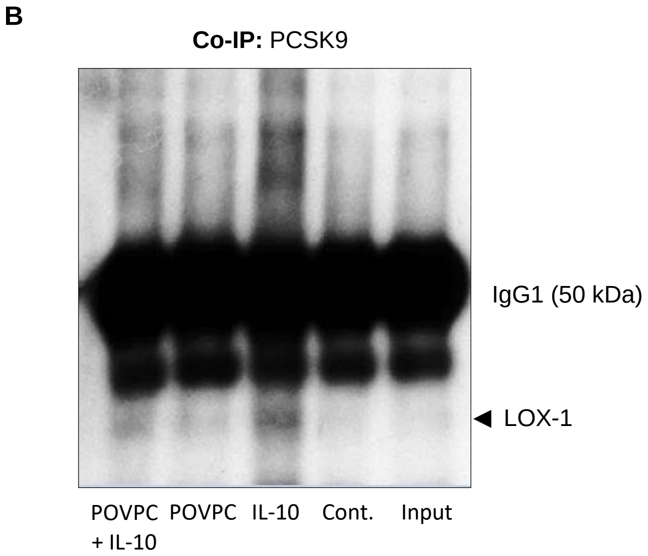

Supplementary Fig 2: Interaction of PCSK9 and LOX-1: **A)** Isolated adult rat cardiomyocytes were pre-treated with PCSK9 inhibitor, PEP 2-8 (2µM) for 4h, followed by IL-10 treatment for 18h. Western blots were done using specific PCSK9 and LOX 1 antibodies. GAPDH was used as a loading control. **B)** Interaction of PCSK9 with LOX1 demonstrated by co-immunoprecipitation using PCSK9 antibody followed by immunoblotting with LOX1. Representative images are shown here from two independent experiments.

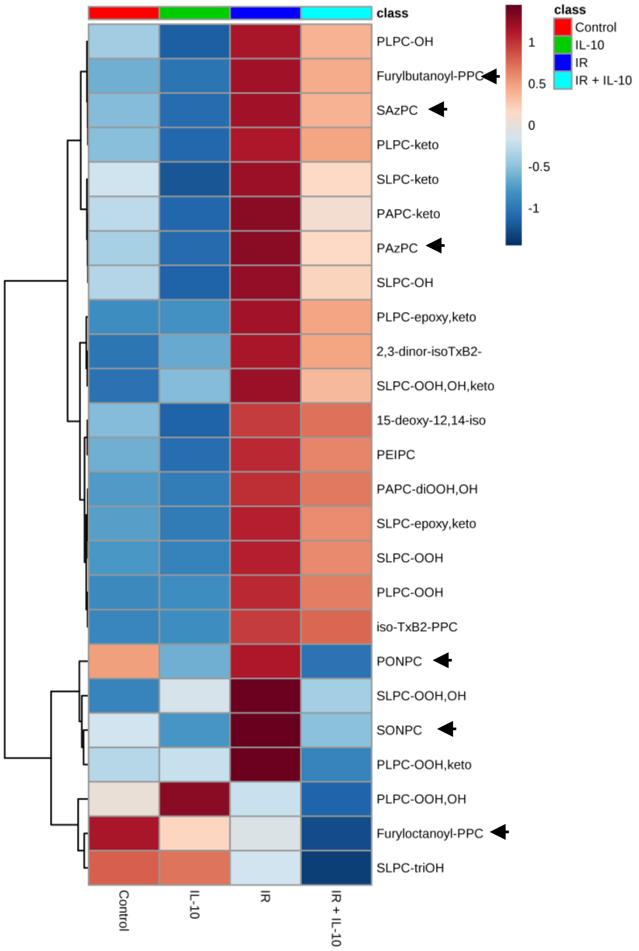

Supplementary Fig 3: Heatmap for fragmented and non-fragmented OxPCs at $\log_2$ fold change. Arrowheads indicate fragmented OxPCs that are maximally affected by IL-10 treatment.

LOX 1 and GAPDH : Manuscript Fig 2A-i

LOX-1

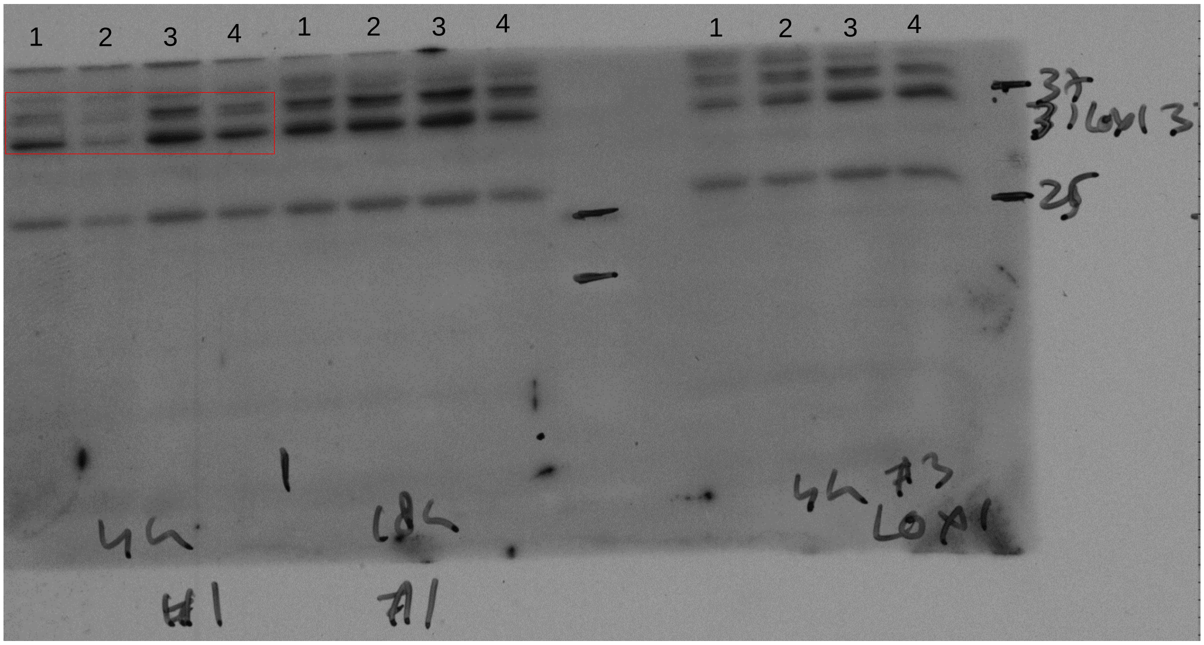

GAPDH

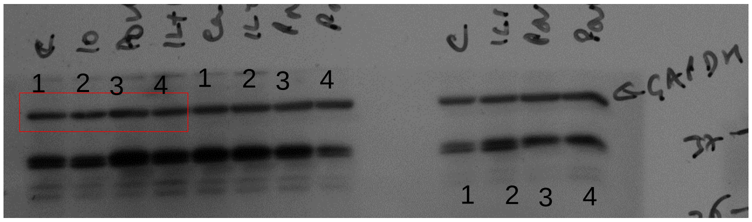

4h **18h** **4h**

Original Immunoblots for figure 2A - i used in the manuscript for Lox 1 (**upper panel**) and GAPDH (**lower panel**). Red rectangles are the images cropped for 4h and presented in the manuscript. Lane 1 = Control (c); Lane 2 = IL-10; Lane 3 = POVPC and Lane 4 = POVPC + IL-10.

Note: This image also contains the another run for 4h and 18h.

TLR2 **TLR2 and GAPDH: Manuscript Fig 3A**

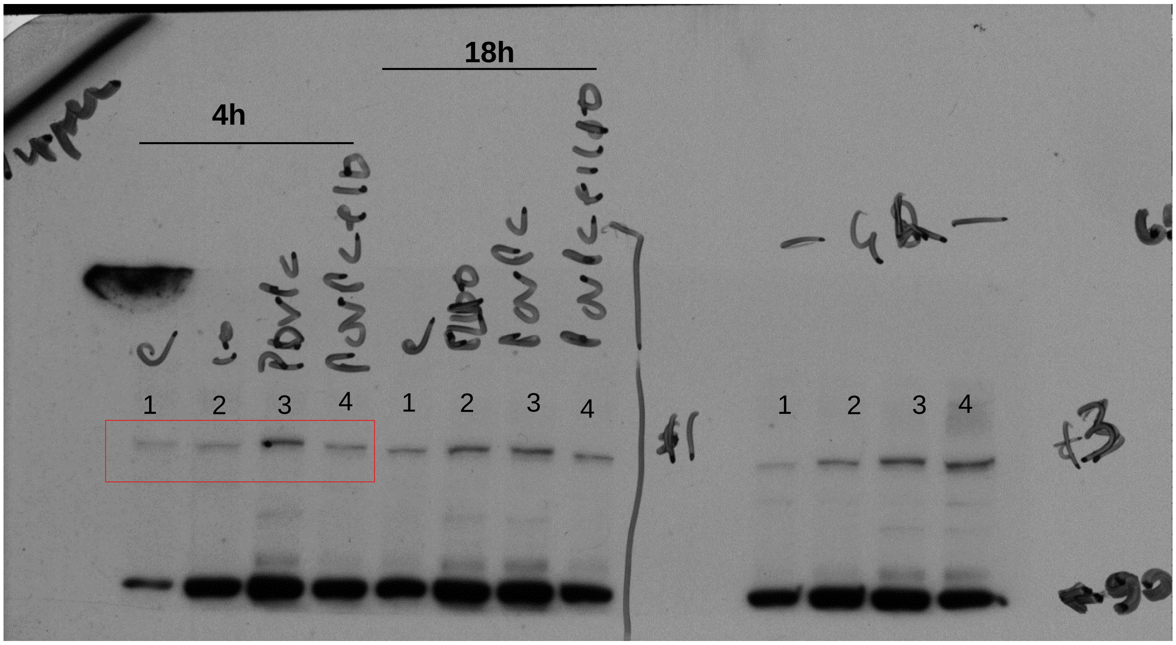

GAPDH

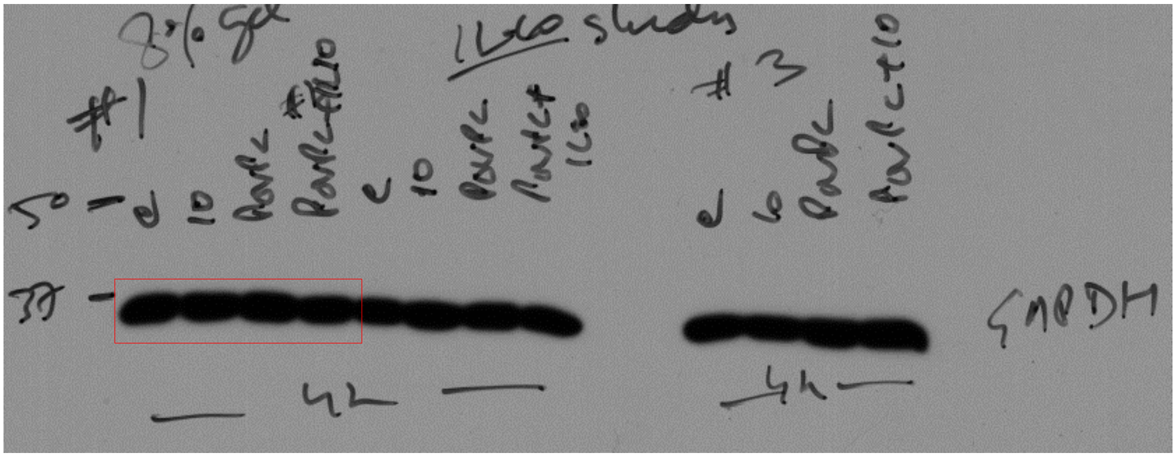

Original Immunoblots for figure 3A used in the manuscript for TLR2 (**upper panel**) and GAPDH (**lower panel**). Red rectangles are the images cropped for 4h and presented in the manuscript. Lane 1 = Control (c); Lane 2 = IL-10; Lane 3 = POVPC and Lane 4 = POVPC + IL-10

PCSK9

PCSK9 and GAPDH: Manuscript Fig 3B- 4h and 18h

A

Gel #1

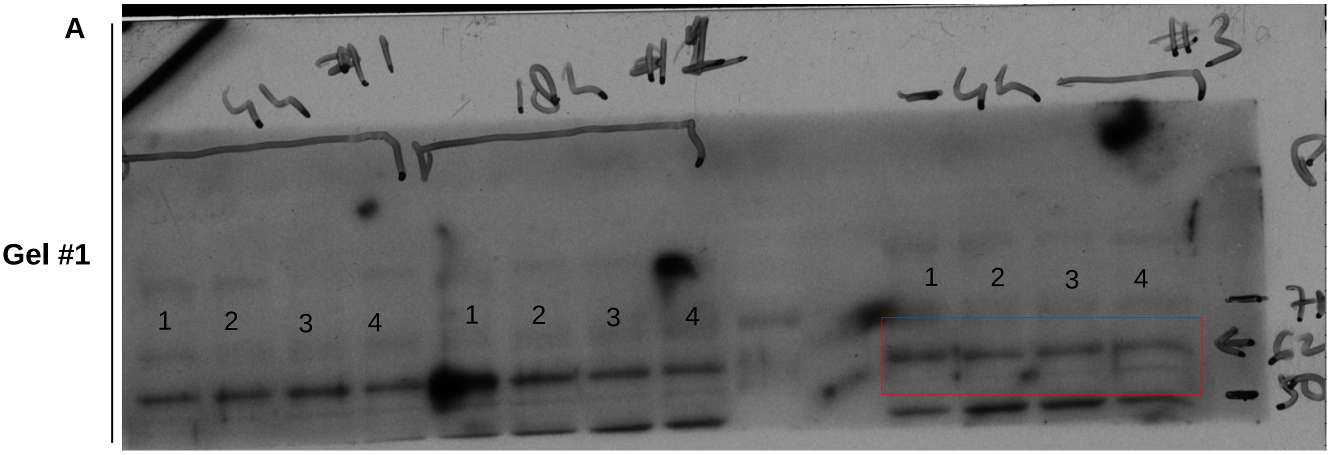

B

Gel #2

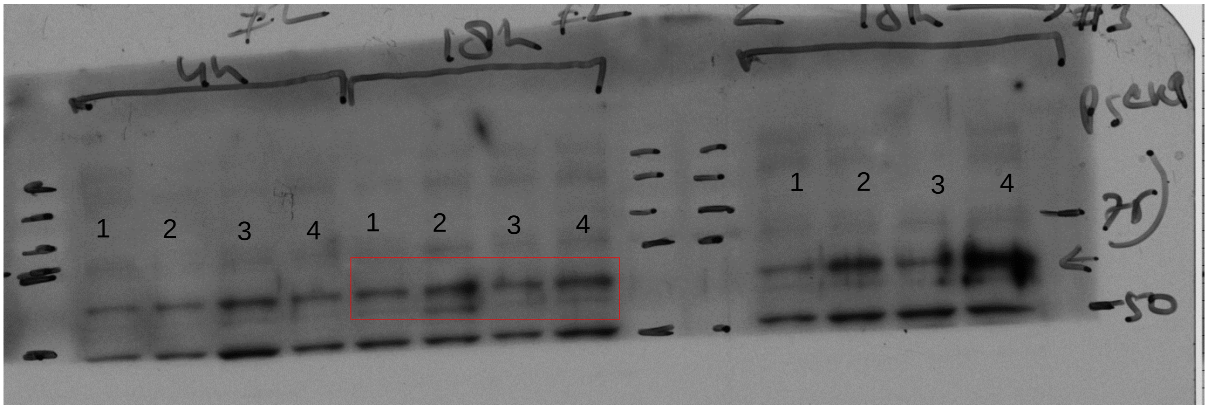

GAPDH **Gel #1** **Gel #2**

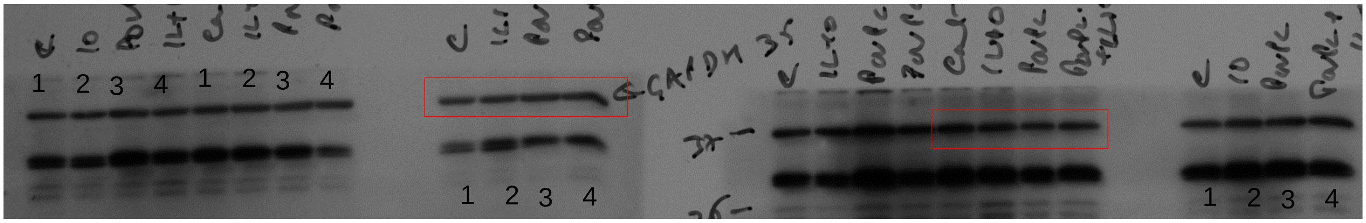

4h **18h** **4h** **4h** **18h** **18h**

Original Immunoblots for figure 3B used in the manuscript. **Upper panel**: PCSK9 (**A and B**) and GAPDH (**lower panel**) for 4h (A) and 18h (B). Red rectangles are the images cropped from each blot that are shown in the manuscript. Lane 1 = Control (c); Lane 2 = IL-10; Lane 3 = POVPC and Lane 4 = POVPC + IL-10

SREBP 1c and GAPDH: Manuscript Fig 3C

SREBP-1c

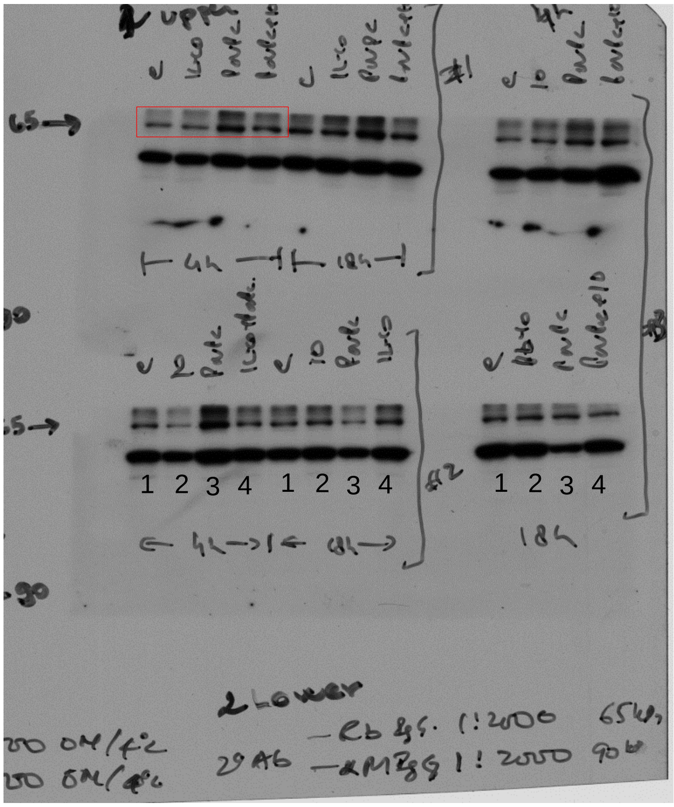

Gel #1

Gel #2

GAPDH

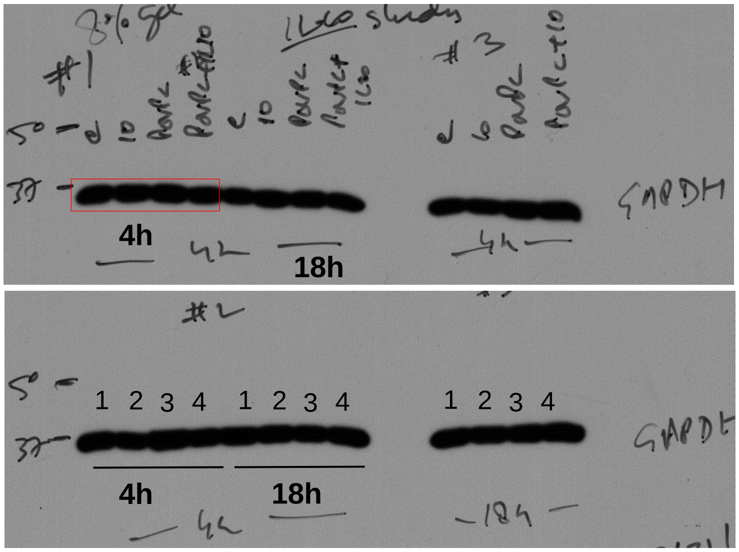

Gel #1

Gel #2

Original Immunoblots for figure 3C used in the manuscript. SREBP-1c (**upper panel**) and GAPDH (**lower panel**). Red rectangles are the images cropped from each blot that are shown in the manuscript. Lane 1 = Control (c); Lane 2 = IL-10; Lane 3 = POVPC and Lane 4 = POVPC + IL-10

Troponin 1c and GAPDH: Manuscript Fig 4A-i

Troponin -1c

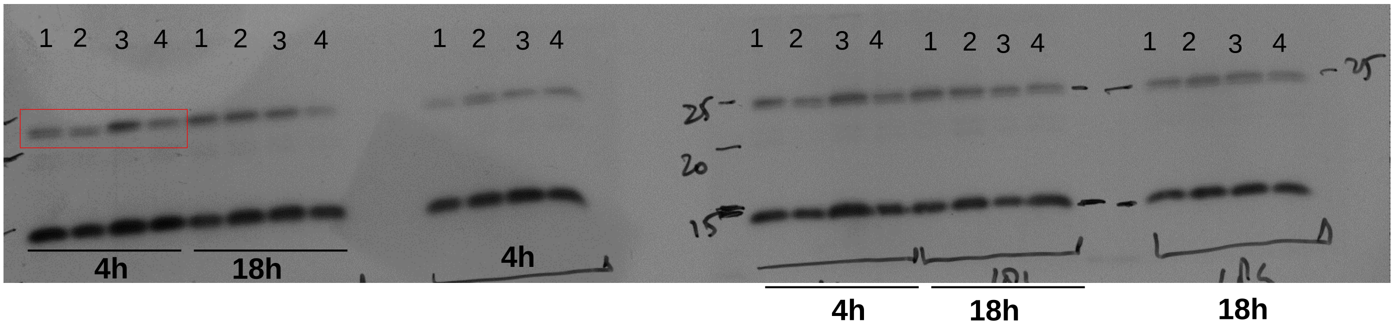

GAPDH

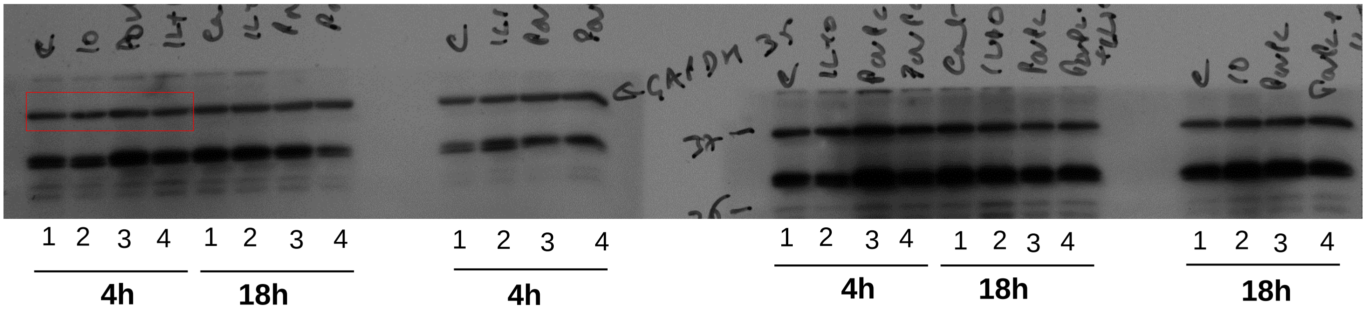

Original Immunoblots for figure 4A-i used in the manuscript. Troponin-1c (**upper panel**) and GAPDH (**lower panel**). Red rectangles are the images cropped from each blot that are shown in the manuscript. Lane 1 = Control (c); Lane 2 = IL-10; Lane 3 = POVPC and Lane 4 = POVPC + IL-10

Lox-1 and GAPDH: Manuscript Fig 5C

LOX-1

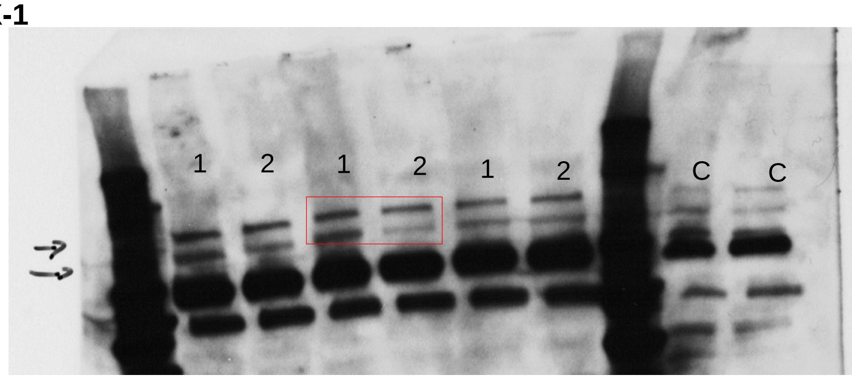

GAPDH

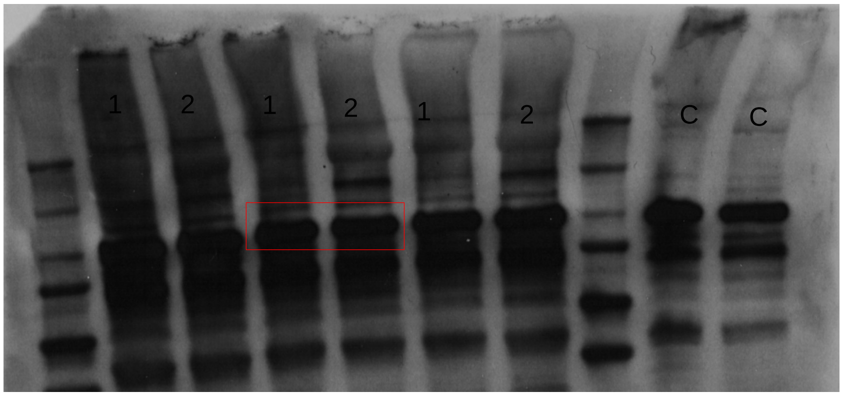

Original Immunoblots for figure 5C used in the manuscript. Lox-1 (**A**) and GAPDH (**B**). Red rectangles are the representative image crops for each protein expression that are shown in the manuscript. Lane 1 = I/R; Lane 2 = I/R + IL-10 and Lane C = Control.

p-SREBP 1c and GAPDH: Manuscript Fig 5E

p-SREBP-1c

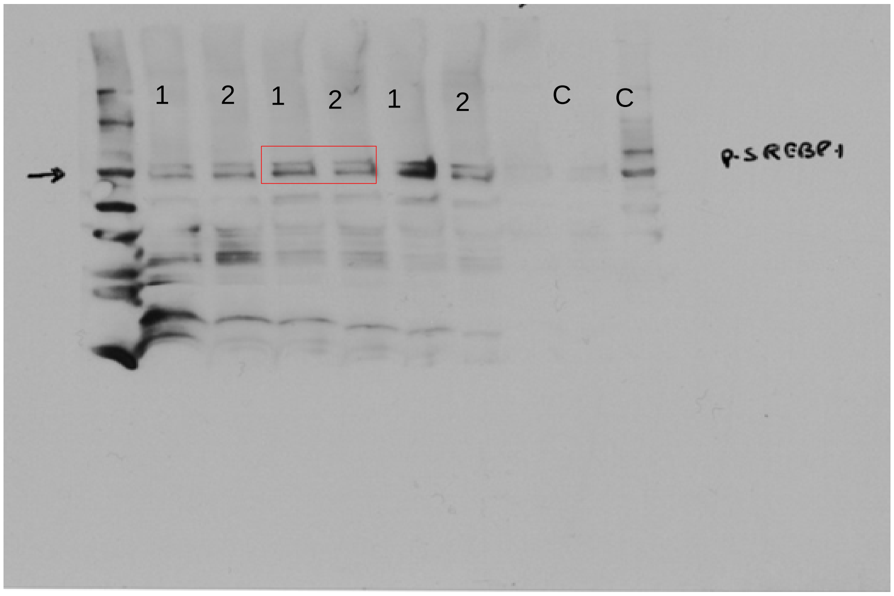

GAPDH

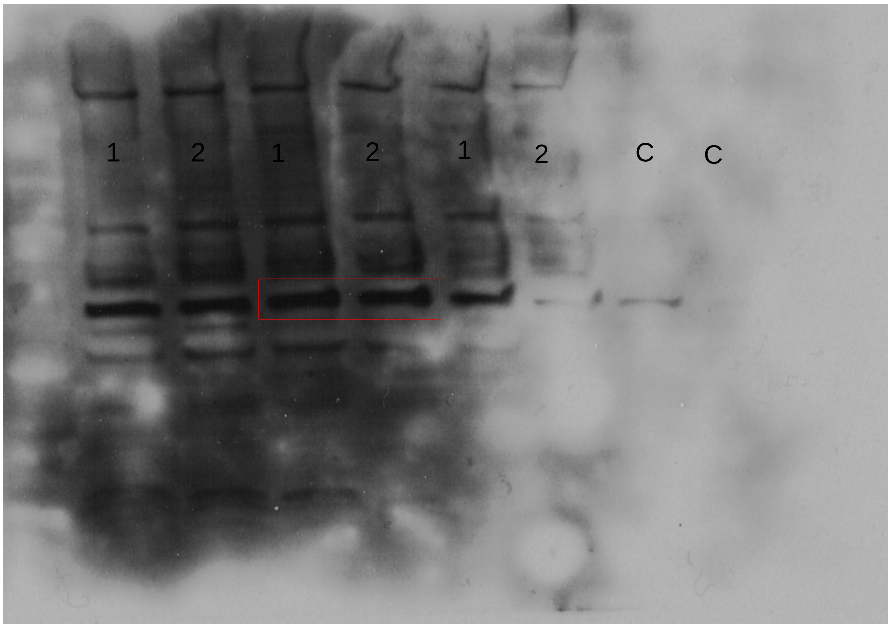

Original Immunoblots for figure 5E used in the manuscript. SREBP 1c (**upper panel**) and GAPDH (**lower panel**). Red rectangles are the representative image crops for each protein expression that are shown in the manuscript. Lane 1 = I/R; Lane 2 = I/R + IL-10 and Lane C = Control.

PCSK9, LOX-1 and GAPDH: Supplementary Fig 2

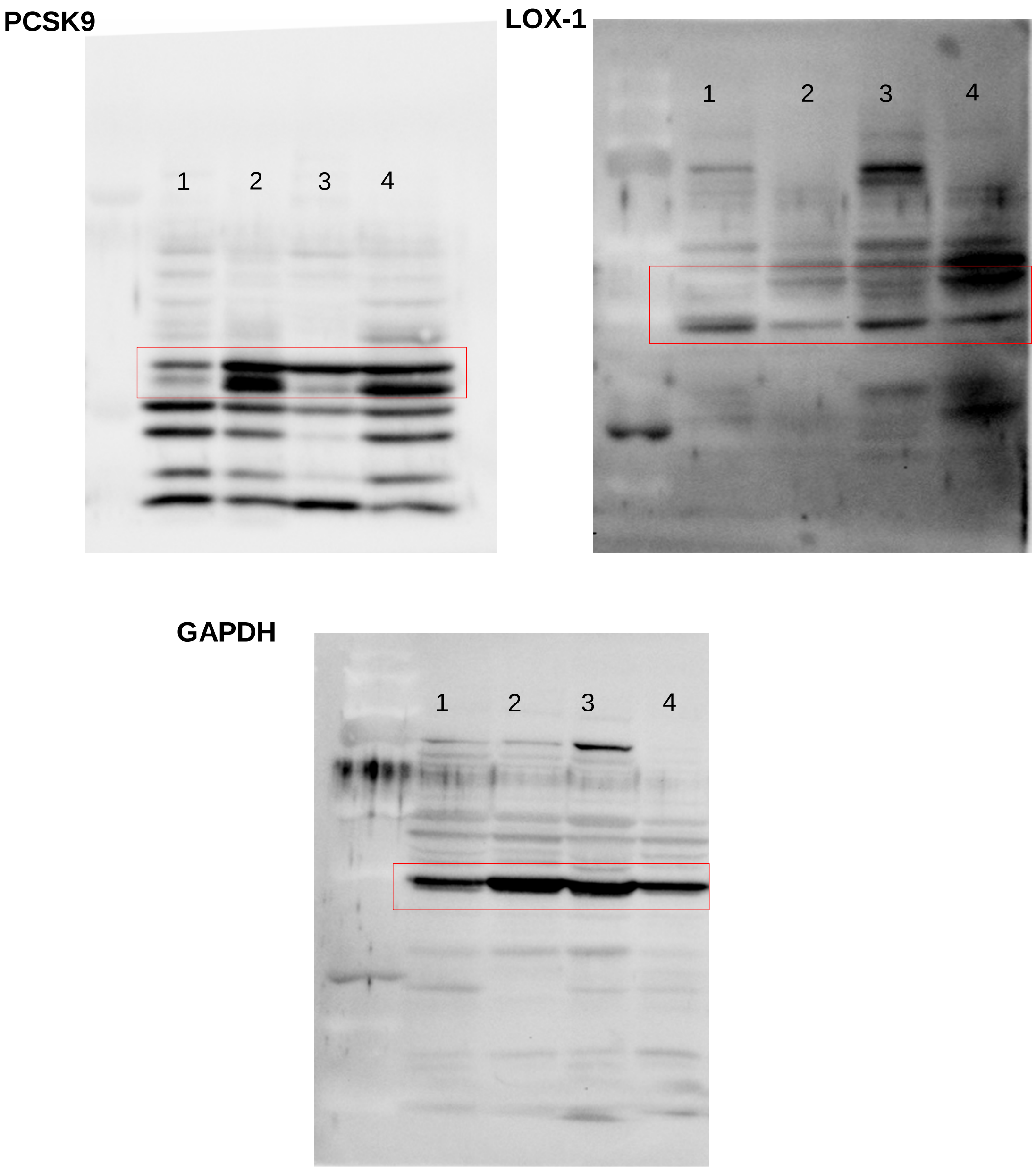

Original Immunoblots for supplementary figure 2A used in the manuscript. PCSK9 (**upper right panel**), LOX-1 (**upper left panel**) and GAPDH (**lower panel**). Red rectangles are the images cropped from each blot that are shown in the manuscript. Lane 1 = Control (c); Lane 2 = IL-10; Lane 3 = PCSK9i and Lane 4 = PCSK9i + IL-10

PCSK9 and LOX-1 interaction : Supplementary Fig 2B

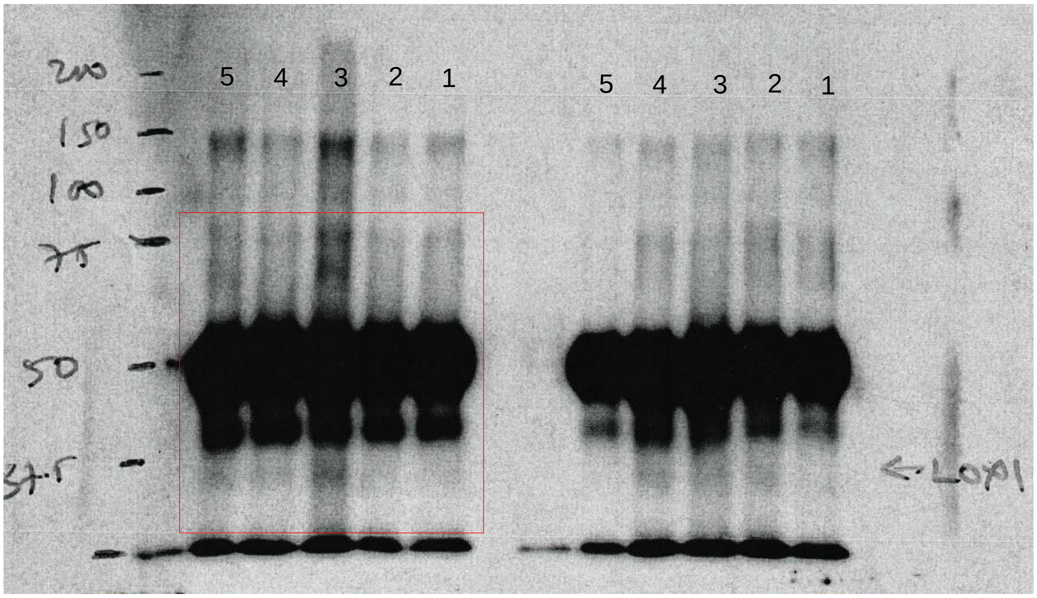

Original Immunoblots for supplementary figure 2B used in the manuscript. Red rectangles are the images cropped from each blot that are shown in the manuscript. Lane 1 = Input (Ab control); Lane 2 = Control; Lane 3 = IL-10; Lane 4 = POVPC and Lane 4 = POVPC + IL-10
